# Quantum Dots for Live Cell and *In Vivo* Imaging

**DOI:** 10.3390/ijms10020441

**Published:** 2009-02-03

**Authors:** Maureen A Walling, Jennifer A Novak, Jason R. E Shepard

**Affiliations:** University at Albany, Department of Chemistry, 1400 Washington Ave., Albany, NY 12222, USA; E-Mails: mw7018@albany.edu (M. W.); jn876455@albany.edu (J. N.)

**Keywords:** Quantum dots, *in vivo* imaging, applications, biocompatibility, nanoparticles

## Abstract

In the past few decades, technology has made immeasurable strides to enable visualization, identification, and quantitation in biological systems. Many of these technological advancements are occurring on the nanometer scale, where multiple scientific disciplines are combining to create new materials with enhanced properties. The integration of inorganic synthetic methods with a size reduction to the nano-scale has lead to the creation of a new class of optical reporters, called quantum dots. These semiconductor quantum dot nanocrystals have emerged as an alternative to organic dyes and fluorescent proteins, and are brighter and more stable against photobleaching than standard fluorescent indicators. Quantum dots have tunable optical properties that have proved useful in a wide range of applications from multiplexed analysis such as DNA detection and cell sorting and tracking, to most recently demonstrating promise for *in vivo* imaging and diagnostics. This review provides an in-depth discussion of past, present, and future trends in quantum dot use with an emphasis on *in vivo* imaging and its related applications.

## 1. Introduction

Fluorescence techniques are commonplace in life science applications, but are constantly evolving as novel optical reporters and imaging instrumentation continues to be developed. Specific conventional biological applications employing fluorescence include cellular and molecular imaging, cell labeling and tracking, multiplexed analyses, and DNA detection. The broad applicability of fluorescence studies, as well as the constant desire to perform more sensitive or advanced measurements, has driven the development of more versatile, reliable fluorescent reporters useful for both single- and multi-color experiments. An ideal fluorescent marker for single-color applications should be bright, biocompatible, non-toxic to the sample, and be stable against photobleaching; in multi-color experiments, fluorophores should exhibit these same characteristics as well as have spectrally resolvable emission profiles with narrow, symmetric emission spectra. In addition, for simplicity and cost benefit, a preferable experimental protocol employing fluorescent reporters should be amenable to relatively inexpensive instrumentation, such as the need for a single excitation source.

Standard fluorescent labels, such as organic dyes, can be problematic, or even limiting, particularly in multicolor experiments due to issues associated with signal intensity strength, relatively short lifetimes, narrow excitation ranges, and broad emission spectra. These problems exist due to the optical properties of organic fluorophores and the growing requirement for more sensitive, complex imaging experiments within the visible spectrum. For example, most optical reporters have specific absorption ranges that prohibit single-source excitation of multiple dyes. In addition, these fluorophores generally have broad emission spectra that create overlapping detection ranges, making multiplexed analysis and data interpretation more challenging. To address these limitations, more robust reporter species are continually being developed. Development of new reporter species and more sensitive instrumentation has lead to continued improvements in biological imaging and analysis. One such reporter moiety that has enabled important advancements in imaging and analysis is semiconductor nanocrystals, also called quantum dots. Quantum dot nanoparticles display many improved optical qualities desirable for biological applications, and are advantageous for both single- and multi-color experiments as compared to organic dyes [1,2].

**Figure 1.**
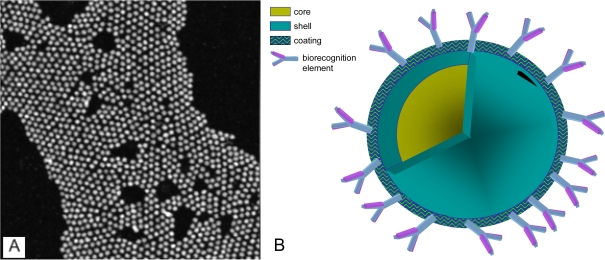
(A) Scanning transmission electron micrographs of PbSe quantum dots: low resolution (full scale = 320 nm) showing the ordering of an ensemble of nanocrystals. Reprinted with permission from [18]. (B) Schematic of a quantum dot conjugate used for in vivo imaging. The image shows a cross section where the quantum dot semiconductor core is visible, which is coated with a shell material, comprised of a second semiconductor material. The core/shell quantum dots are coated for biocompatibility and solubility, followed by conjugation to a biological element for recognition or a specific interaction in vivo. In this instance, the quantum dot bioconjugate is shown covered with antibodies. For illustrative purposes, and not drawn to scale.

Quantum dot nanoparticles generally fall within the 2–10 nanometer size range and possess size-tunable, i.e. controllable, optical and electrical properties (Table 1). The most commonly used quantum dots fall within this size range, but larger nanoparticles have also been fabricated and used for other specific applications. The optical properties of quantum dots stem from their composition: a semiconductor material core, such as cadmium selenide, lead selenide, or indium arsenide (CdSe, PbSe, or InAs), passivated by a coating, or shell, also of semiconductor material (Figure 1) [1–6]. The core semiconductor material has a narrow bandgap, defined as the minimum amount of energy required to excite an electron from its ground state to the next highest energy level. The core material is enclosed within a shell coating, comprised of a different semiconductor material of higher bandgap. The higher bandgap energy associated with the shell effectively limits, or confines, the excitation and emission solely to the core material. This excitation of the quantum dot core occurs through absorption of energy, which causes an electronic transition from the ground state to the first excited state. Absorption is followed by the release of energy in the form of a photon when the electron relaxes back to the ground state. The core-shell architecture not only confines excitation and emission to the core, but also enhances the photoluminescence quantum yield of the core emission and protects the core from photobleaching [1,4,6,10,11].

The increase in photoluminescence quantum yield results from a surface passivation effect of the core, where the number of nonradiative recombination sites, such as holes and gap states, are reduced, enhancing the charge transfer [4,6,21,22]. Quantum dots have been estimated to be up to 20 times brighter and 100 times more stable than traditional fluorescent reporters [2]. The increased stability and emission intensities of quantum dots makes them more practical in ultrasensitive detection, long term imaging, and rapid-detection applications, such as flow cytometry [2,23–26].

**Figure 2.**
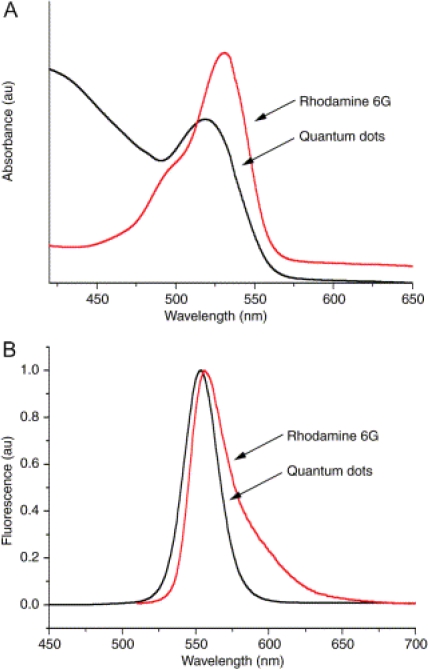
Comparison of (A) the excitation and (B) the emission profiles between rhodamine 6G (red) and CdSe quantum dots (black). The quantum dot emission spectrum is nearly symmetric and much narrower in peak width. Its excitation profile is broad and continuous. The quantum dots can be efficiently excited at any wavelength shorter than ∼530 nm. By contrast, the organic dye rhodamine 6G has a broad and asymmetric emission peak and is excited only in a narrow wavelength range. Reprinted with permission from [40].

**Figure 3.**
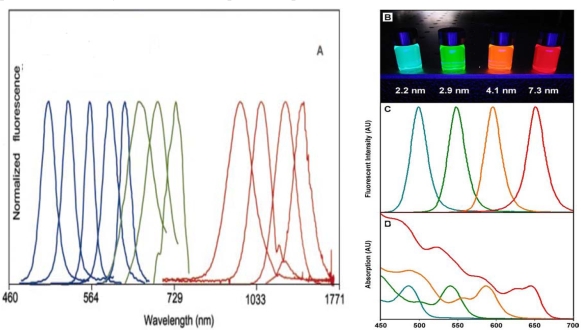
(A) Size- and material-dependent emission spectra of several surfactant-coated semiconductor nanocrystals. The blue series represents different sizes of CdSe nanocrystals with diameters of 2.1, 2.4, 3.1, 3.6, and 4.6 nm (from left to right). The green series is of InP nanocrystals with diameters of 3.0, 3.5, and 4.6 nm. The red series is of InAs nanocrystals with diameters of 2.8, 3.6, 4.6, and 6.0 nm. (B, C, D) Size-dependent optical properties of cadmium selenide quantum dots dispersed in chloroform, illustrating size tunable fluorescence emission. (B) Fluorescence image of four vials of monodisperse quantum dots with sizes ranging from 2.2 to 7.3 nm in diameter, obtained with ultraviolet lamp illumination at 365 nm. (C) Fluorescence spectra of the same four quantum dot samples, excited at 400 nm. Narrow emission bands (23–26 nm full-width at half-maximum) indicate narrow particle size distributions. (D) Absorption spectra of the same four samples. Notice that the onset of absorption is slightly blue-shifted from the emission peak for each quantum dot sample, and that the absorption spectra are very broad, so that a wide spectrum can be used for excitation. Both the absorption and emission intensities are plotted in arbitrary units (AU). Adapted with permission from [1,48].

Along with an increased quantum yield, the size-tunable nature and narrow emission profiles associated with quantum dots are major advantages that stem from their unique core/shell architecture Quantum dot emission profiles contain characteristic peaks at wavelengths across the visible spectrum that are independent of excitation wavelength (Figures 2 and 3) [1,3,7,17,19,20]. Although quantum dots exhibit these numerous advantageous optical properties, inorganic semiconductor materials are toxic to living systems, effectively limiting their use in biological systems. While nanoparticle toxicity continues to be an area of much research and debate [27–31], numerous experiments have documented modified quantum dots as having limited cytotoxicity, and surface coatings have been developed in an effort to minimize toxicity [24–26,30–39]. More detail regarding optical properties and quantum dot toxicity will be discussed in later sections of this review.

Quantum dots, despite their many advantages, have additional issues that must be considered prior to their widespread adoption, including intermittent on/off behavior, biocompatibility, and a larger relative size as compared to standard fluorophores. These limitations will be discussed briefly, but all have been managed to some extent and are continually being studied, improved and addressed. First, the fluorescence signals of individual quantum dots display intermittent on/off behavior, or “blinking”, which can complicate fluorescence measurements or be problematic in ultrasensitive analysis [2,41]. Issues with blinking have generally been overcome by increasing the shell size and/or decreasing the excitation intensity [42,43]. Second, as quantum dots are comprised of inorganic materials, they are incompatible with polar solvents, and have limited utility in biological applications without further derivatization. Substantial effort has gone into developing methods, such as different coatings or caps, to render quantum dots soluble in aqueous solution [1,2,32,35,44–46]. Finally, since quantum dots are an order of magnitude larger than standard organic dyes, quantum dots are less applicable when the fluorescent probe’s size must be minimized [26,45]. Quantum dot size is not considered an experimental hindrance, but is worth noting, because these nanoparticles fall in the size range of macromolecules, such as most proteins and peptides, etc. [35,45–48]. Conjugation and biocompatibility are all critical factors for *in vivo* imaging, and both topics will be discussed in later sections.

**Figure 4.**
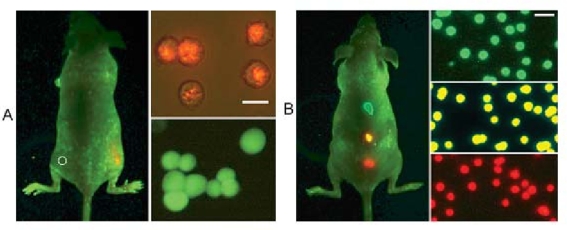
Sensitivity and multicolor capability of QD imaging in live animals. (A,B) Sensitivity and spectral comparison between QD-tagged and GFP transfected cancer cells (A), and simultaneous *in vivo* imaging of multicolor QD-encoded microbeads (B). The right-hand images in A show QD-tagged cancer cells (orange, upper) and GFP-labeled cells (green, lower). Approximately 1,000 of the QD-labeled cells were injected on the right flank of a mouse, while the same number of GFP-labeled cells was injected on the left flank (circle) of the same animal. Similarly, the right-hand images in B show QD-encoded microbeads (0.5 μm diameter) emitting green, yellow or red light. Approximately 1–2 million beads in each color were injected subcutaneously at three adjacent locations on a host animal. In both A and B, cell and animal imaging data were acquired with tungsten or mercury lamp excitation, a filter set designed for GFP fluorescence and true color digital cameras. Transfected cancer cell lines for high level expression of GFP were developed by using retroviral vectors, but the exact copy numbers of GFP per cell have not been determined quantitatively. Reprinted with permission from [23].

**Figure 5.**
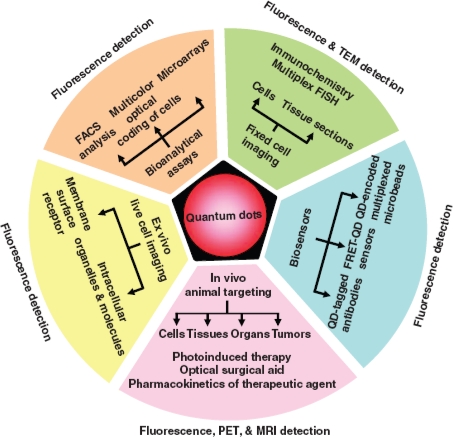
Applications of quantum dots as multimodal contrast agents in bio-imaging. Reprinted with permission from [61].

Quantum dot properties are controllable and their signal strength is robust, and as such, are desirable for a wide range of *in vivo* studies and labeling applications (Figures 4 and 5), immunoassays, optical encoding for multiplexed analyses, cell tracking, and fluorescence resonance energy transfer (FRET) [1,2,24–26,32,34–37,41,45–47,49–58]. Even though quantum dots have demonstrated their value in numerous applications, their physical characteristics continue to be refined to further their utility. Synthetic methods, including conjugation and aqueous solubilization steps, are critical to maximizing quantum dot properties for broader use. The next few sections will detail past and ongoing efforts to address these issues and expand the opportunities in quantum dot research.

## 2. Quantum dot synthesis

Although semiconductor nanoparticle syntheses were reported in the 1980’s, the first protocol producing quantum dot samples that were highly monodisperse, regular in core structure, size-tunable, and with surface capping was reported by Bawendi and coworkers in 1993 [3,59,60]. Numerous modifications have since been made to these seminal procedures, improving or changing the properties of quantum dots to better suit specific experimental needs. These alterations to standard synthesis parameters have produced quantum dots with varying composition, size (emission wavelength), shape, and shell, among others. Despite the many synthetic permutations and their modifications, quantum dot synthesis for biological applications has four basic steps: core synthesis, shell growth, aqueous solubilization, and biomolecular conjugation.

### 2.1. Core synthesis

Quantum dot cores are composed of semiconductors of group II–VI (CdSe, CdS, CdTe), group IV–VI (PbS, PbSe, PbTe, SnTe), and group III–V (InP), or any other combination with extremely narrow bandgaps, the most common being CdSe [1,3,4,7,13,15–20]. In a general synthetic method, organometallic liquid precursors are injected into hot (290°–350°C) coordinating solvents, such as trioctylphosphine oxide (TOPO) and trioctylphosphine (TOP). Coordinating solvents stabilize the bulk semiconductors and prevent aggregation as the quantum dots grow [3,11]. After the desired growth time is reached in relation to size and optical properties, aliquots are removed from the reaction mixture, cooled, and purified. Purification steps, such as precipitation in anhydrous methanol or butanol, are performed and can serve as size exclusion steps to ensure uniformity [3,7,13]. The uniformity and average nanocrystal size can be affected by temperature differences of less than 1°C [13]. If suitable conditions for injection temperature and growth time are maintained during synthesis, separate size-selection steps are not necessary to achieve a narrow size distribution [13,18]. Nanoparticles synthesized in this manner result in the semiconductor core surrounded by TOPO, in a nonpolar solvent such as chloroform.

The central synthesis methodology to provide protocols that allow quantum dots with customizable properties is now relatively straightforward and reproducible. Through the many modifiable parameters involved with synthesis (precursors, solvents, reagent concentrations, temperature, growth time, etc.), quantum dots of varying core compositions and sizes can be produced, resulting in specific synthetic methods that correlate directly to the desired optical properties (Table 1). Common experimental variations include the alteration of the core composition by the use of different metallic precursors, or additional reagents in the solvent mixture, such as hexadecylamine (HDA), which improves monodispersity and eliminates size exclusion steps [3,7,9,12,15–20]. Ultimately, to fabricate quantum dots of varying particle size (and varying emission wavelengths) in a single reaction, one-pot synthesis can be carried out in non-coordinating solvents, with aliquots quenched at time points throughout the reaction. Each quenched aliquot results in a specific diameter of nanoparticle with relatively uniform distribution [8,14].

### 2.2. Shell growth and surface modification

The semiconductor core material must be protected from degradation and oxidation to maintain and optimize quantum dot performance. Both shell growth and surface modification enhance stability and increase photoluminescence of the core [1,3–6,11,22]. As mentioned previously, shell growth provides protection by coating or capping the core with a thin layer of a second semiconductor material with a higher band gap. The semiconductor shells are also inorganic in nature, commonly employing compounds such as zinc sulfide or zinc selenide (ZnS, ZnSe). Surface protection can also be achieved through modification of the core, which is carried out in organic solvents, such as alkylamines, but including a semiconductor shell layer is most common protection method [3,11].

Semiconductor shells are grown epitaxially by addition of shell precursors drop-wise into a crude nanocrystal mixture at a temperature below that of core synthesis [4,22,60]. The reduced temperature facilitates shell growth by capping the pre-existing nanoparticle cores instead of initiating a separate nucleation of shell precursors. Shell thickness is controlled by varying the growth temperature and the concentration and rate at which reagents are added [6,22]. If the shell growth temperature is too close to that of the core synthesis temperature, core seeds will continue to grow and negatively effect (broaden) the size distribution. Conversely, if the shell growth proceeds at lower temperatures, the crystallinity of the shell is decreased, leading to imperfect passivation of the core surface, among other problems [6,22,60]. The concentration of shell precursors and their rate of addition are crucial to promote heterogeneous growth. Shell formation and thickness is measured in a series of discrete monolayers, which for ZnS shells are ∼3.0–3.5 Å thick, but again vary based on the shell composition and its growth method. The number of shell monolayers is controllable, but generally does not exceed more than five [4,6,11,22,44,60]. Even when strict precautions are taken, some shell precursor elements may nucleate separately and size selection steps are often required to ensure the quality of the final product. The overall process of epitaxial shell growth results in the formation of core/shell nanocrystals surrounded by the coordinating solvent such as TOPO, used during growth, dispersed in a nonpolar solvent.

In contrast to shell growth, direct surface modifications of the core using organic solvents occur through surface exchange reactions. For example, surface exchange can be performed by mixing crude TOPO-coated nanocrystals with an organic solvent, such as an alkylamine [3,11]. The alkylamine acts as a competing capping group and displaces the TOPO from the core surface. This exchange reaction can take place in a single step, or can be done as a series of steps. With the single step process, the extent of coverage is determined by the length of exposure to the capping group and is measured at the conclusion of the process. The benefit of the multiple step method is more reliable control over the extent of coating. When coating in a stepwise manner, optical measurements taken at each step in the process can measure the positive effect on quantum yield. Core modification by surface exchange reactions is generally reversible, and because of the nature of the capping material, this type of organically-capped quantum dot cores cannot be employed in biological applications [11].

The most common cap for CdSe nanocrystals is a ZnS shell. The ZnS shell does not incorporate into the core or alter the core structure, but has been shown to increase the quantum yield of the CdSe core by 50–66% [4,6,11,52]. Slightly smaller increases have been observed with organic alkylamine caps, demonstrating an increase in the core quantum yield by up to 50% [3,11]. Both shells and caps also provide important protection, contributing to the stability of the core against degradation and photobleaching. Finally, the addition of a shell or cap material can also provide terminal functional sites to provide for further derivatization, such as aqueous solubilization or bioconjugation.

### 2.3. Aqueous solubilization

The inorganic core-shell semiconductor nanoparticles, once prepared, are soluble in nonpolar solvents only. To have utility in biological applications, nanoparticles must be soluble in aqueous solutions and require surface modifications to achieve biocompatibility. Two general approaches have been used to achieve aqueous solubility: surface ligand exchange and amphiphilic polymer coatings.

#### 2.3.1. Surface ligand exchange

**Figure 6.**
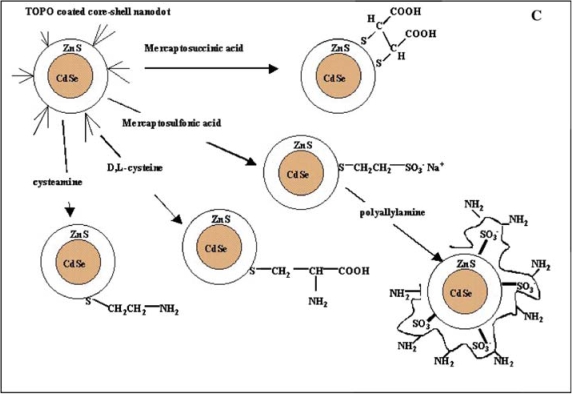
Schematic illustration of CdSe/ZnSe nanocrystals possible solubilization methods and stabilization procedure. Reprinted with permission from [52].

Two different surface ligand exchange approaches can be used for solubilization. The first method involves exchanging the coordinating ligands (e.g. TOPO) on the quantum dot shell surface. The exchange process is similar to surface exchange reactions of the core described previously, and optimally results in the addition of a heterobifunctional ligand. A bifunctional ligand employs a hydrophobic end to displace the TOPO from the quantum dot, while a hydrophilic end extends out into solution, aiding in solubility. Thiol groups are a common functionality employed to link to the shell surface, but this functional group can detach from the quantum dot surface in a reversible fashion [1,62–64]. Successful thiol group attachment has been achieved using mercapto-compounds, cysteine residues, or chemically reduced proteins [44,47,51,65,66]. In each of these cases, a carboxyl group or protein residues served as the hydrophilic tail, extending out into solution and allowing the quantum dots to be soluble in an aqueous environment (Figure 6).

**Figure 7.**
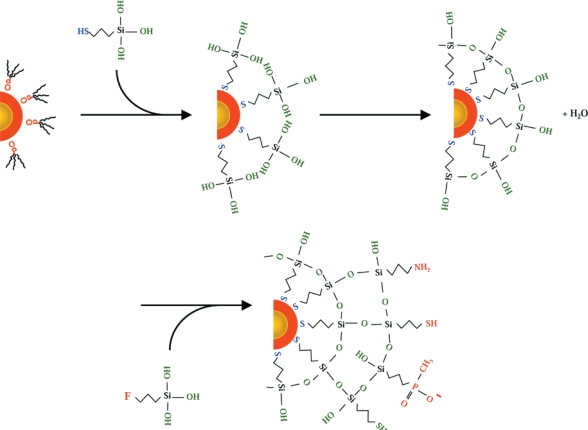
Sketch of the silanization method. The TOPO-capped CdSeZnS core/shell particles are dissolved in pure MPS, drawn in its hydrolyzed form for convenience in the figure. After basification, the MPS replaces the TOPO molecules on the surface. The methoxysilane groups (Si-OCH_3_) hydrolyze into silanol groups (Si-OH), and form a primary polymerization layer. Heat strengthens the silanol-silanol bridges by converting them into siloxane bonds and releasing water molecules. Then, fresh silane precursors containing a functional group (F = −SH, −NH_2_; −PO−(O−)CH_3_) are incorporated into the shell and may tailor the nanocrystal surface functionality. In a last step (not shown) the remaining hydroxyl groups are converted in methyl groups; this last step blocks further silica growth. Reprinted with permission from [62].

The second surface exchange method involves silane derivatives, used to displace the coordinating ligand on the quantum dot surface, and eventually resulting a layer of silica around the quantum dot (Figure 7) [1,62]. The reaction conditions, in particular reaction time, contribute to the thickness of the silica shell. Silica shell growth around a core/shell quantum dot involves mixing the quantum dots with a compound at basic pH over several days while continually heating, cooling, and washing the solution. Silica shell growth requires multiple purification steps. Compared with the synthesis of mercaptoacetic acid-capped quantum dots, encapsulating quantum dots with a silica shell is more complicated, but the difficult nature of synthesis is countered by its advantages. This latter approach is advantageous because quantum dots coated with silica are more stable due to the high degree of cross-linking between the silane molecules. This extensive cross-linking ensures solubility even if some thiol groups are lost [62]. An additional advantage of using silica shell coatings is that the procedures do not change if a different type of siloxane is used. Ultimately, using either surface ligand exchange approach for solubilization leaves quantum dots susceptible to aggregation and precipitation in biological buffers, and may require additional efforts to reduce or minimize aggregation [2,26,38,39,45,62].

#### 2.3.2. Amphiphilic polymer coatings

Alternately, core/shell semiconductor quantum dots can be coated with an amphiphilic polymer, such as octylamine-modified polyacrylic acid [32,34,35,46]. This approach utilizes the nonpolar quantum dot shell for interaction with the hydrophobic portion of the polymer, allowing the hydrophilic portion of the polymer to increase solubility. Growing an amphiphilic polymer shell around quantum dots is similar to coating with silica, but instead of forming the shell by displacing the TOPO molecules left on the surface during synthesis, the amphiphilic polymer takes advantage of the hydrophobic nature of the coordinating ligands. The interactions associated with this type of solubilization are similar to those in micelle formation.

### 2.4. Conjugation

For biological applications, quantum dots must be linked to biomolecules without altering the biological activity of the conjugated form. A number of successful conjugation methods have been developed, including covalent and non-covalent attachment methodologies. Specific conjugation methodologies include direct adsorption on the quantum dot surface, the use of inert polymer coatings, or biotin-streptavidin linkages.

Covalent attachment of biomolecules to quantum dots is achieved through direct linkage to the quantum dot surface coating or via small molecule cross-linkers. During synthesis, silica, silane derivatives, or other coatings can include functional groups capable of direct conjugation [1,23,26,32,62]. Such cross-linking strategies exploit the functional groups present on both the quantum dot surface and the biomolecule. For example, carbodiimide compounds are commonly used to link amino-functionality with carboxyl-groups. Covalent attachment is a simple, effective way of linking biomolecules to quantum dots and contributes minimally to the overall bioconjugate size. Another common conjugation scheme employs the biotin-streptavidin linkage, which requires coupling of the quantum dot to streptavidin. Quantum dot-streptavidin conjugates are useful because a wide range of proteins and other biomolecules can be biotinylated. These conjugates have applications in staining and labeling [1], live tracking, and drug screening [35,41,53].

Simple electrostatic interactions have been employed for non-covalent biomolecular attachment since most water solubilization methods result in carboxylic acid coatings, which are negatively charged under biological conditions [1]. For example, quantum dots capped with dihydrolipoic acid (DHLA) can be bound electrostatically to positively charged proteins and used in imaging experiments [25] or detection assays [54]. While electrostatic interactions are less stable than covalent attachment, quantum dot-bioconjugates formed in this fashion can be used for many of the same applications [25,44,54,65]. Direct adsorption of a biomolecule to a quantum dot surface is another non-covalent attachment method [45,47,51]. Chemically modified peptides and other biomolecules have been shown to adsorb spontaneously on the surface of water-soluble CdSe/ZnS quantum dots. For example, quantum dots coated with adsorbed peptides have been used for *in vivo* imaging to locate cells with specific surface proteins [51] or different types of vasculature in tumors or organs [45].

For the majority of applications, quantum dots act only as nonfunctional probes and have minimal impact on the experiment, the binding event, or the surroundings. Nonspecific attachment to unintended molecules and aggregation of quantum dot-conjugates is possible, and if so, could negatively impact the results of an experiment. Coating quantum dots with an inert hydrophilic polymer, such as polyethylene glycol (PEG), minimizes or eliminates these problems [45,46]. The conjugation method should not interfere with the optical properties of the quantum dots or the activity of the biomolecule. However, work continues to be done to characterize linking designs and offer improvements [67].

## 3. Properties

A major reason quantum dots have found such utility in the life sciences is their unique properties, which can be optimized to meet the specific needs of a wide range of experiments. As discussed in the introductory sections, the relevant advantageous properties include controllable emission wavelengths, sharp emission profiles, robust signal strength, and the use of a single excitation source. The optical properties can be influenced by varying different aspects of the quantum dots, all of which can be controlled, including core size, core composition, shell composition, and surface coating. While all the aforementioned qualities influence quantum dot emissions, the core size and composition have the most influence over the range of the emission spectra (Table 1; Figure 3). Varying either the size of the quantum dot core or its composition can result in a customizable emission profile with a specific maximum anywhere across the electromagnetic spectrum, starting in the ultraviolet (UV) region and including the near-infrared (near-IR) region [3,7,17,19,20]. Altering the shell composition and/or surface coating affects the stability of the core and results in increased photoluminescence, but does not significantly affect the emission range. Emission spectra for semiconductor nanoparticles are distinctive, containing narrow and symmetric peaks independent of the excitation energy, as long as the excitation energy is greater than that of the band gap energy [1,4]. This characteristic means quantum dots of varying sizes and compositions can be excited with a single source. However, the relative intensities of different quantum dot emission profiles will vary with excitation wavelength independently of one another (Figure 3D), based on their quantum efficiency at that wavelength. Finally, because quantum dot emission peaks are substantially narrower than those of organic dyes, more quantum dot emissions are resolvable within the visible spectrum than possible with standard fluorophores [1,2,68].

## 4. Aqueous solubilization

For applications in biological systems, an additional hydrophilic coating must be included after quantum dot core/shell synthesis to render quantum dots soluble in aqueous environments. While only two general mechanisms are used for quantum dot solubilization, surface ligand exchange and coating with an amphiphilic polymer, a wide range of molecules are available to serve as surface coatings to solubilize quantum dots, including thiolate ligands, silica, and various polymers.

### 4.1. Thiolate ligands

Thiolate ligands have proven especially useful in solubilizing quantum dots with a ZnS shell because the sulfur from the ligand binds to the zinc of the shell coating with high affinity. When a thiolate ligand contains a second hydrophilic functional group, such as a carboxylic acid or amine, the ligand sulfur binds to the quantum dot shell and the hydrophilic group extends into solution for increased solubility. Quantum dots coated with thiolated ligands employ a surface exchange reaction between the new thiolated ligand and the coordinating ligand remaining on the surface from particle synthesis [1,2]. Ligands of varying lengths can be employed to reduce or eliminate passive adsorption of proteins and other biomolecules. The optical properties of quantum dots with thiolated coatings are comparable to the original core/shell nanoparticle, with high quantum yields, narrow emission spectra, and increased photostability [2,25,44,45,47,50,65]. An example of this method applied to overcome early solubilization issues was the research of Nie and coworkers, who used mercaptoacetic acid as a biocompatible coating for sensitive cellular labeling and detection [2]. Since then, a wide number of other carboxylic acid-thiolate ligands have been used, including mercaptopropionic acid (MPA) and DHLA [25,29,44,65].

**Figure 8.**
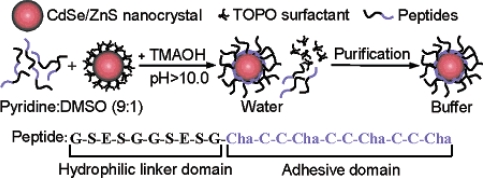
Schematic representation of the surface coating chemistry of CdSe/ZnS nanocrystals with phytochelatin-related R-peptides. The peptide C-terminal adhesive domain binds to the ZnS shell of CdSe/ZnS nanocrystals after exchange with the TOPO surfactant. A polar and negatively charged hydrophilic linker domain in the peptide sequence provides aqueous buffer solubility to the nanocrystals. TMAOH: Tetramethylammonium hydroxide; Cha: 3-cyclohexylalanine. Reprinted with permission from [51].

An alternate method for quantum dot solubilization employs modification with a coating of cysteine residues [66]. Quantum dots coated with a layer dl-cysteine residues were available for use in cellular and fixed-tissue imaging applications [52,66]. Similarly, cysteine-rich, amphiphilic peptides have also been used as water-soluble coatings for quantum dots (Figure 8) [51]. The amphiphilic peptides used for biocompatibility contained a hydrophilic domain for aqueous solubilization and a cysteine-rich ‘linker domain’ to bind to the quantum dot surface. When cysteine residues in the linker domain were replaced with alanine residues, the resulting particles were water-insoluble, which demonstrated that the thiol group of cysteine is necessary for solubilization. Most all of these coating methods offer terminal functional groups that provide the possibility of further conjugation, and cysteine-quantum dot conjugates were successfully linked to biotin and used to label avidin-conjugated cell-surface proteins on live cells [51].

While sulfur and zinc bind with high affinity, the bond holding most thiolate ligands to the quantum dot surface is dynamic, meaning quantum dots solubilized using thiolate ligands will have a some degree of shelf life and can be susceptible to aggregation in biological solutions [62–64]. Therefore, additional steps are necessary to reduce aggregation and increase stability. Incorporating inert polymers in the surface coating along with the thiolated ligand has proven useful to both increase stability and decrease aggregation. Examples of polymers increasing quantum dot stability and shelf life include PEG, which has decreased aggregation by replacing some ligands on the quantum dot surface [45], and poly(allylamine), which has increased the stability of dl-cysteine-coated quantum dots for fixed-cell imaging [52].

### 4.2. Silica

The Alivisatos group published seminal work that showed quantum dots could be encapsulated in a silica shell for water solubility [1]. The same group later elaborated on these methods to demonstrate functionalization and applicability in live cell imaging [26,62]. Modified siloxanes with varying functional groups are available for conjugation, which allows tailored coatings and encapsulation protocols, while providing an added layer of protection. This added layer both protects the quantum dot against degradation and photobleaching, while also decreasing the toxicity to the surrounding environment by preventing or minimizing the release of core materials. Quantum dots coated with polymerized silica shells have greater stability prior to conjugation, because the extensive polymer network provides additional stability [1,62].

### 4.3. Polymers

Other polymeric materials have been employed in solubilization techniques, taking advantage of the various chemical compositions and charges available. As mentioned previously, amphiphilic polymers are a common choice, because they simultaneously exploit the hydrophobic nature of the quantum dot surface for binding and the aqueous environment for solubility [35,46]. The difference between coating quantum dots with a siloxane polymer and an amphiphilic polymer is the method of encapsulation: silica coatings displace the hydrophobic coordinating ligand on the quantum dot surface, while amphiphilic polymers utilize it for binding. As with other methods of solubilization, the optical properties of the original quantum dots are not adversely affected by an added polymer layer. While the additional polymerized layer increases the stability in buffer solutions, any increase in size has the potential to hinder biological activity [32,35].

**Figure 9.**
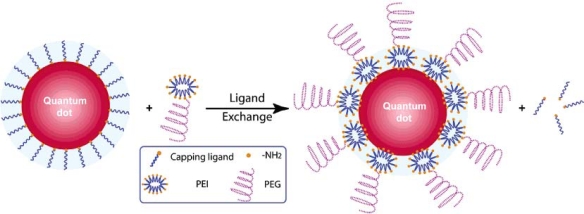
Schematic diagram showing direct exchange reactions between the monovalent capping ligand octadecylamine and the multivalent copolymer ligands. Reprinted with permission from [39].

Polymers are useful functional coatings because they can be modified to contain such groups as thiols or amines to aid in further conjugation. For example, an amine-modified polymer, such as octylamine-modified polyacrylic acid, can be used to conjugate quantum dots to biomolecules with a carboxyl group using standard cross-linking molecules [35]. The polymer coating charge can be altered by varying the functional groups contained in the polymer, which can be useful for specific applications such as entry to the intracellular space [30,39]. Finally, polymer compositions are critical to optimize solubility and limit other undesirable characteristics of the quantum dots, such as aggregation and toxicity (Figure 9) [32,39].

A specific type of amphiphilic polymer, PEG-incorporated phospholipid micelles, has been used to encapsulate quantum dots for *in vivo* cell-lineage tracking [32]. The quantum dot-micelles remained in *Xenopus* embryos until the tadpole stage, were resistant to photobleaching, and showed no signs of cellular toxicity. During cell division, the original labeled cells retained some quantum dots, while some were passed to daughter cells. In this manner, cell lineage could be traced by measuring the fluorescence intensity within individual cells without fear of photobleaching affects compromising the study. In hybridization experiments, quantum dots encapsulated with micelles and then coated with single-stranded DNA did not interrupt the hybridization to complementary DNA. Quantum dot-micelles were found to be stable in buffer solutions containing salt and showed no obvious signs of aggregation [32].

## 5. Cytotoxicity

When determining biocompatibility of quantum dots, solubility is not the only consideration; biocompatibility also requires limited toxicity to biological systems, such as interruption to the cell’s normal activities. The quality and specific degree of quantum dot toxicity has been extensively explored. For example, toxicity has been directly related to oxidation of the nanoparticle core/shell material, leading to the release of free cadmium. The nature of the surface coatings obviously plays an important part of this process, and this aspect, among others, will be discussed in relation to cytotoxicity.

### 5.1. Oxidation and cadmium release

Because quantum dot cores are made with elements that are inherently toxic to cells and living systems, concerns exist over their potential toxicity for *in vivo* applications. Cadmium ions have been shown to bind to thiol groups on critical molecules in the mitochondria and cause enough stress and damage to cause significant cell death [27,69]. Cadmium is the most widely used material for quantum dot cores, so its release into the cell would be a logical mechanism of toxicity. Derfus *et al*. used hepatic cells to monitor toxic effects of quantum dots, as the liver is the primary site for acute damage from cadmium and a major accumulation site for nanoparticles [27]. Results indicated that oxidation of the nanoparticle surface, either induced by exposure to air before solubilization or catalyzed by UV light, caused oxidation of selenium and/or sulfur, exposing free cadmium [3,6,13,70]. Exposure to air before solubilization or moderate to prolonged exposure to UV light after incubation increased the amount of cadmium in the cells enough to cause observable cell death. Further efforts demonstrated that cells labeled with quantum dots synthesized under stable, inert conditions showed no toxic effects. In addition to air and UV exposure, quantum dot surface oxidation can occur in oxidative solutions, such as hydrogen peroxide, which is relevant because such environments are possible *in vivo*. In a separate report using CdSe/ZnS core/shell nanoparticles solubilized with mercaptoacetic acid, Kirchner *et al*. showed that Cd^2+^ was released into cells at toxic levels over time (48 hours), under standard synthesis and imaging conditions [29]. By examining the effects of the free core materials in solution, cadmium was determined as the primary cause of cytotoxicity, but its levels could be reduced or eliminated by adding additional surface coatings [27,29].

### 5.2. Surface coatings

Prevention of core material oxidation is essential to reducing toxicity and increasing biocompatibility of quantum dots. Coatings used to increase the quantum yield of the core, or for solubility and conjugation, have been shown to reduce cytotoxicity of quantum dots. The fundamental notion is that additional layers act as a physical barrier to the core, preventing access, with different surface coatings having varying levels of passivation.

A common surface shell coating for CdSe core-quantum dots is ZnS, and as mentioned, the additional semiconductor layer increases the material’s photoluminescence [4,6]. Additionally, the semiconductor shell aids in reducing the cytotoxicity of the core material, as the concentration where cell death was first observed was approximately nine times higher for CdSe/ZnS (core/shell) quantum dots than with CdSe core quantum dots alone [29]. The ZnS shell protects the core from oxidation and other environmental factors that contribute to cadmium release. The shell materials are also prone to oxidation, but to a lesser extent than the core materials [27], because zinc and sulfur bind with high affinity and form a polymer-like structure around the core [4,6]. Oxidation due to air exposure before solubilization was nearly eliminated when quantum dots contained a ZnS shell, as shell oxidation was observed only with prolonged exposure to UV light [27]. When the sulfur was oxidized, the ZnS shell-network was disrupted, leaving the core materials exposed, resulting in similar oxidation as seen when the ZnS shell does not fully cover the core [6,27]. Without the disruption of the shell, the environment has no interaction with the cadmium of the core, and it therefore can not be toxic to the system. Ultimately, oxidation of the shell material leads to increased toxicity due to resultant oxidation of the core and release of cadmium.

Charged surface coatings have been used both for solubility and conjugation to quantum dots, but can also have the added benefit of reducing toxicity. Ligands with terminal carboxylic acid, hydroxyl, or amine groups (such as 11-mercaptoundecanoic acid, thioglycerol, and amine-PEG, respectively), have been used for solubilization because they bear a negative charge at biological pH. These quantum dot conjugates have all been the focus of studies regarding cytotoxicity. Terminal carboxylate coatings have proven to be the least effective at preventing core oxidation, cell death, and inflammatory responses [27,28,38], and quantum dots with hydroxyl-terminated ligands demonstrated only slightly better toxicity effects [28]. As these surface ligands are often thiolated for conjugation with the zinc shell, in acidic or oxidative conditions, the interaction between zinc and sulfur can be broken and the surface ligand released [1].

While the disruption of this interaction could conceivably be used for applications like delivery of molecules into cells, the disruption of the coating is problematic for two reasons. First, some ligands themselves have proven toxic in a number of biological systems [28,29]. Second, the absence of a surface ligand leaves the core/shell exposed and can lead to oxidation and/or decreased solubility. In cytotoxicity studies monitoring the inflammatory response in skin cells, PEG-amine-coated quantum dots caused little to no release of pro-inflammatory cytokines and insignificant cell death [38]. In particular, the quantum dots with amine-terminal ligands showed less cytotoxicity than other ligands. This decreased cytotoxicity could also be contributed to the added protection of the PEG layer and not the amine ligand, as PEG-modified silica has proven exceptionally stable and almost completely eliminated any cytotoxicity [27,29,31,38]. PEG-quantum dots demonstrated extremely low levels of toxicity, even at high concentrations (up to 30 μM), but also diminished uptake by cells as compared to other surface coatings [29]. Amphiphilic polymers and larger biomolecules, such as bovine serum albumin (BSA), have been used to coat quantum dots, providing a physical barrier to the core similarly to silica shells [27,29]; the physical barrier provided by larger polymers and biomolecules shields the environment from the toxic elements of the core and is only effective when the coating remains undisturbed. Coatings of this type had a tendency to accumulate on the cell surface, possibly contributing towards cell death, which further illustrates that cadmium release is not the only cause of concern when dealing with quantum dots [29]. Regardless of the coating is used, quantum dot cytotoxicity decreases with increasing numbers of surface layers, and coating must remain intact to provide sufficient protection. For biological applications, simply adding more layers to quantum dots to eliminate cytotoxicity is not an infallible strategy, because the surface coatings must be tailored to meet the specific needs of the experiment, and eventually, size can become an issue.

### 5.3. Genotoxicity and cell activity disruption

Cell death caused by cadmium ion release is not the only type of toxicity exhibited by nanoparticles. Quantum dots can also damage DNA and disrupt normal cell activity caused by factors such as the surface coatings themselves. Zhang *et al*. made phenotype and whole genome measurements of human fibroblast cells exposed to PEG-silica-coated quantum dots, as they were proven to be the least toxic and most stable. The fibroblast cells were monitored for other activity interruptions based on exposure to nanomolar quantum dot concentrations [31]. Minimal statistically relevant effects were observed in the cell cycle as significant alteration in expression levels (defined as more than 2-fold expression levels) occurred in only ∼50 genes total, or about 0.2% of all genes studied. These results demonstrated that PEG-silica coated quantum dots did not induce a strong response correlating to heavy metal-related toxicity or strong anti-inflammatory response. Other separate studies concerning surface coatings and cytotoxicity probing the activity of different cells confirmed this work and showed no interruption or disruption in activity or function [27–29]. However, in a separate study by Hoshino and coworkers, DNA damage was observed based on interactions with quantum dots coated with carboxylic acids [28], so while the field of cytotoxicity continues to develop, much research in the area is currently ongoing and will be of much future interest. Because of the limited information on quantum dot cytotoxicity and the continued ongoing research, broad generalizations regarding quantum dot cytotoxicity should be forestalled, until conflicting reports on surface coating protection and degradation, core oxidation, cell activity disruption, and toxicity are reconciled and additional conclusive studies have been performed and validated.

## 6. Applications

Quantum dots have properties that provide advantages beneficial for a number of different life science applications as compared to the standard reporters current employed. The improved brightness and photostability exhibited by quantum dots are justification for their increased use in imaging and labeling experiments. The ability to render quantum dots biocompatible and non-toxic extends their applicability to *in vivo* vasculature imaging and tracking. The robustness of their signal strength also affords utility in targeting and detection applications. Their simple, routine fabrication protocols and uniform spectral profiles are now allowing quantum dots to realize their full potential, as quantum dot applications are branching out into high throughput, multiplexed analyses and quantitative analysis of biomolecules *in vivo*. These applications will be discussed in detail in the following section.

### 6.1. In vivo Targeting and Imaging

*In vivo* applications are especially problematic for any reporter species due to the need to optimize the numerous parameters associated with imaging animals, tissue, or live cells. Such applications require many of the most advantageous optical properties exhibited by quantum dots, but also necessitate biocompatibility, low toxicity, proper attachment of biomolecules, and navigation of the cascade of events involved in the immune response. The following seminal works address each of these issues to demonstrate the full capabilities of quantum dots.

#### 6.1.1. Targeting

The work by Schroder *et al*. demonstrated that quantum dots can target specific receptors *in vivo* [71]. Quantum dots were conjugated to folate, a critical nutrient necessary for rapid growth and cell division, to perform assays targeting the folate-specific receptor. TOPO-coated quantum dots (CdSe) were prepared in phospholipid micelles and assessed in animal studies. The folate-quantum dot conjugates were specifically detected at the folate receptors in mouse lymphoma cells after incubation for two hours. An increase in fluorescence intensity over non-specific quantum dots in the same cell line was observed, demonstrating that folate was the main factor in bio-recognition and was highly specific in its targeting. As folate is critical for cell growth, the folate receptor will have higher expression levels in cancer cells than in normal cells. The determination of the extent of folate receptor expression could then be a possible diagnostic tool, as any significant intensity increase as compared to normal expression levels is an indication of over-expression, and may be important to cancer diagnosis.

Antibody-quantum dot conjugates were also used to optimize circulation times and provide specificity for *in vivo* applications [72]. Noteworthy research by Jayagopal *et al*. used these conjugates for standard microscopy determinations, flow cytometry assays, and *in vivo* imaging. The quantum dot conjugates targeted cell adhesion molecules related to retinal vasculature in rats in a multiplexed fashion using a single excitation source. The researchers employed a PEG crosslinking scheme to link the antibodies and were able to discriminate between different cell adhesion molecules by conjugating specific monoclonal antibodies to quantum dots. This work demonstrated noninvasive, *in vivo* imaging of the retinal vasculature, while providing the spatial resolution down to the level of single cells. The fluorescence intensities increased within 30 minutes, whereas non-specifically labeled quantum dots and the control showed no fluorescence localization in the vasculature.

#### 6.1.2. Imaging

A specific advantage of quantum dots for *in vivo* applications is their photostability; quantum dots allow images to be recorded over a longer period of time than available with the use of fluorescent dyes or proteins due to their resistance to photobleaching. Maysinger *et al*. visualized CdSe and CdTe quantum dots detectable at one hour-, one day-, three days-, and seven days post intracortical injection using *in vivo* imaging techniques [73]. Mice were injected subcutaneously and scanned for fluorescence, in particular in the brain, where peak fluorescence was observable at three days post injection and persisted for seven days. Sub-cellular resolution was achieved and allowed the identification of the location of the conjugates. This work had numerous important features. The researchers demonstrated internalization of their quantum dot conjugates, with varied rate and efficacy of internalization for different cell-types. Neuronal internalization is especially challenging, and *in vivo* imaging in neural cells has important relevance to quantum dot toxicity, as this approach would allow the investigation of the neuronal immune response in real time. This approach was novel not only in the *in vivo* imaging aspects but the animal model was transgenic, and was monitored for an astrocyte-responsive luciferase reporter in addition to the injection of quantum dot conjugates. The activation of the astrocyte response was observable as an increase in bioluminescence in response to foreign nanoparticles in mice.

The work of Jiang *et al*. demonstrated the ability of quantum dots with near-IR region emission wavelengths for *in vivo* analysis of deep tissue or non-invasive applications. The application of near-IR reporters minimized the absorption and scattering of light by native tissues, and allowed the researchers to employ longer wavelengths for a diagnostic emission window, generally between 650 – 900 nanometers [74]. By conjugating transferrin to a quantum dot with an emission at 750 nanometers, *in vivo* observations were performed on mouse heart and femur up to 0.8 millimeters deep beneath the skin. Quantum dot conjugates emitting in the near-IR allowed for greater visualization depth, yielding an increase of up to 800 microns from what was performed previously.

**Figure 10.**
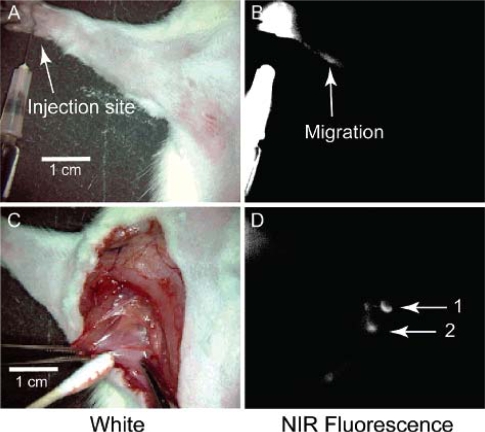
Sequential lymph nodes (1 and 2) and the lymphatic channel between them were imaged (C, D) in a rat by white light and NIR fluorescence five minutes after injection of the quantum dots (A, B). Reprinted with permission from [75].

**Figure 11.**
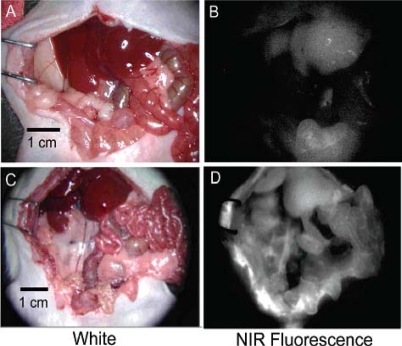
No fluorescence was seen in the interstitial fluid surrounding the incision in the rat model with DHLA-coated (InAs) ZnSe quantum dots (A, B). With DHLA-PEG, however, fluorescence was observed from extravasated quantum dots (C, D). Reprinted with permission from [75].

Zimmer *et al*. have also employed near-IR emitting quantum dots due to their ability to act as a reporter at a wavelength minimally absorbed by biological species [75]. Building off of their previous work, the authors synthesized a series of InAs/ZnSe core/shell quantum dots with a smaller hydrodynamic diameter (less than 10 nanometers) than previously reported. The small core size, along with variation to the shell thickness and composition offered a range of size tunable emission wavelengths, between 750–920 nanometers. The conjugation of DHLA to the quantum dots allowed for the observations within the interstitial fluid in rats, where the quantum dot conjugates exited the blood vessels (Figures 10 and 11). This visualization of the extravasation sites is important, and has potential to interrogate the delivery mechanism of quantum dots to tumor cells. No extravasation was observed in quantum dots without the DHLA coating.

#### 6.1.3. Vasculature imaging

Tumor growth requires a supply of nutrients from the blood stream. Angiogenesis is the process by which new vasculature establishes a blood supply to a growing tumor. Receptors, such as integrin, are highly expressed in tumor cells during angiogenesis, and diagnostics targeting such receptors can lend insight into the type and extent of diseases, including cancer. The work by Cai *et. al*. detailed the use of a tri-peptide-quantum dot conjugate which specifically binds to integrin [76]. The conjugation of arginine-glycine-aspartic acid to a quantum dot with emission at 705 nanometers yielded a diagnostic tool specific for the integrin receptor that emits in the near-IR region. *In vitro* analysis was performed on human glioblastoma and human breast cancer cells, showing specificity for integrin-positive cells only. In addition, binding was inhibited by the presence of an integrin antagonist. These results illustrated that assays were possible with the potential to help differentiate cancer based on integrin expression levels. Injections of both the conjugates and non specific controls through the tail vein of mice also demonstrated the specificity, with a maximum fluorescence at six hours post-injection. This work showed successful *in vivo* tumor imaging by quantum dots, including the extensive vasculature network, and provides an important window into the importance of quantum dots for imaging applications.

The work of Smith *et al*. demonstrated how quantum dots allow for the non-invasive visualization of blood vessel development over time [77]. The conjugation of biotinylated fibrinogen to quantum dots showed specificity towards the membrane of blood vessels during angiogenesis. The conjugates had robust biocompatibility, staying in circulation for days without noticeable toxicity. In addition, residence times could be influenced through alterations of the surface chemistry functionalities. Like work cited previous, the authors employed quantum dots with longer emission wavelengths to avoid native autofluorescence and increase their depth of field. Overall, as directly compared to the standard fluorophore FITC, these quantum dot conjugates imaged vasculature at a comparable intensity level at substantially lower concentrations, almost three orders of magnitude less concentrated.

#### 6.1.4. Tracking

**Figure 12.**
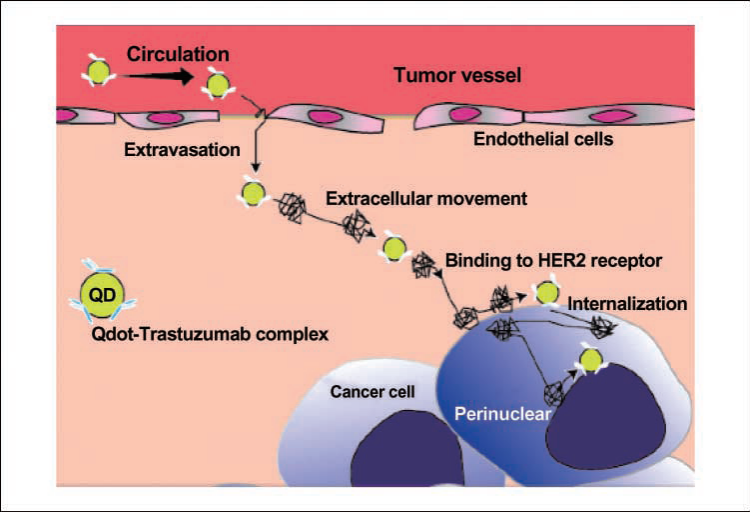
Schematic illustration of the quantum dot complex, the quantum dot complex entered into the circulation, extravasated into the interstitial space from the vascular space, bound to the tumor cells through the interstitial region, and having reached the perinuclear region after traveling on the intracellular rail protein. All processes exhibit a characteristic “stop-and-go” movement. Reprinted with permission from [78].

The work of Tada *et al*. magnificently elucidated the delivery mechanism of quantum dots into human breast cancer cells [78]. The authors employ a cell line that over-expressed the Human Epidermal growth factor Receptor 2 (HER2) on its cell membrane, which is a protein associated with higher aggressiveness in breast cancers. The conjugation of anti-HER2 monoclonal antibodies to quantum dots allowed for the visualization of the nanoparticles in blood vessels serving tumor cells in mice. The quantum dots helped determine the velocity and directionality, among other criteria, for the circulation in the blood vessel, extravasation, binding to HER2 on the cell membrane, and movement into the perinuclear region, etc. (Figure 12). This seminal work allowed the tracking of movements that were random in orientation and speed, exhibited stop and go behavior, and diffused by Brownian motion. This work represents some of the most sophisticated efforts to track biomolecules and understand sub-cellular movements, and will serve as a landmark study in drug delivery.

**Figure 13.**
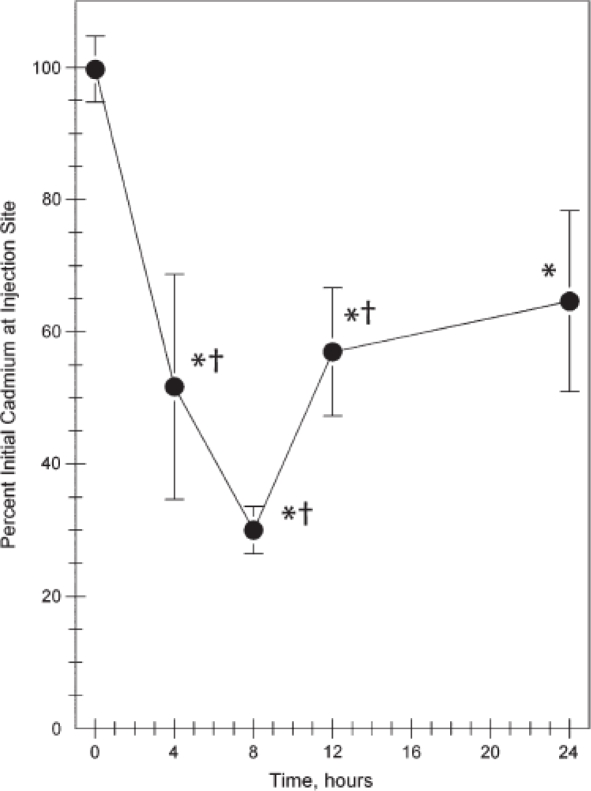
The percent of the initial dose of cadmium that remained at the site of injection of the CdSe quantum dot. The skin that contained the site of injection was removed, completely digested using HNO_3_, and the total cadmium was detected using ICP-MS (mean ± SE). Values indicated by an asterisk (*) were significantly different (p _ 0.05) than the value at 0 h, while values indicated with the cross (†) were not significantly different from each other (p > 0.05). Reprinted with permission from [79].

**Figure 14.**
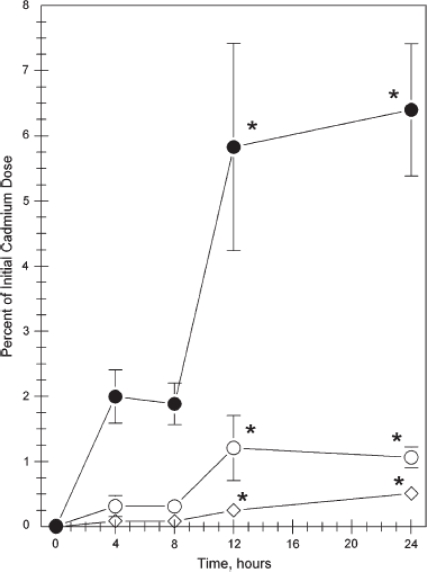
Distribution of cadmium in organs following intradermal injection of CdSe quantum dot. The data are expressed as mean ± SE for: liver (solid circles); regional lymph nodes (open circles); and kidney (open diamonds). Values indicated by an asterisk (*) were significantly different (p < 0.05) than the value in the same organ at 0 h. Reprinted with permission from [79].

The intradermal injection of CdSe/CdS, PEG-coated quantum dots into the right dorsal flank of mice by Gopee *et al*. was performed to monitor the biodistribution by inductively-coupled plasma mass spectrometry (ICP-MS) [79]. The sensitivity of ICP-MS is an enormous asset to identifying trace levels, possibly undetectable by optical methods. ICP-MS was used to quantify the presence of quantum dots in various organs and tissues, such as the liver, spleen, and kidney, among others (Figures 13 and 14). The results showed that the majority of the quantum dot dosage remained at the injection site, but within minutes of injection, traces could be visualized moving from the injection site, presumably via the lymphatic system. The presence of the quantum dots was determined based on cadmium content and verified via fluorescence microscopy, where possible. The PEG coating served as an uncharged, biocompatible surface and allowed the determination of non-specific accumulation of the nanoparticles into the organs involved with the immune response in mice.

**Figure 15.**
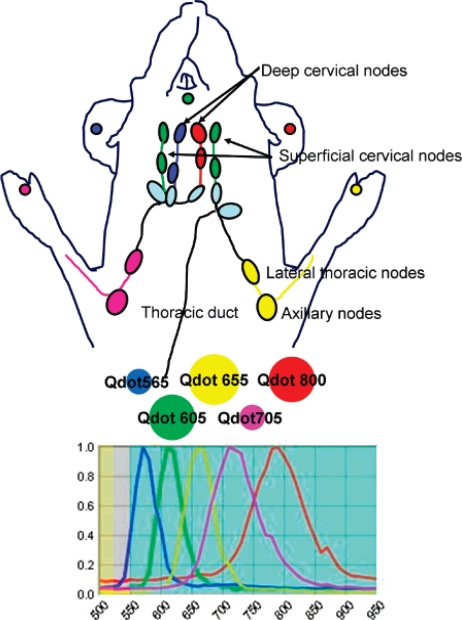
Anatomy of the lymphatic system in the upper body of the mouse and a schematic of five-color spectral fluorescence imaging, with a graph of the emission spectra of each of the five carboxyl quantum dots used (Qdot 565, blue; Qdot 605, green; Qdot 655, yellow; Qdot 705, magenta; Qdot 800, red). The colored lymph nodes are the draining lymph nodes visualized in this study. Reprinted with permission from [81].

The work of Kobayashi *et al*. was an exquisite study of the lymphatic flow by multiplexed analysis using five different quantum dots [80]. This research is the first application of *in vivo* imaging that employs five different reporter quantum dots. Quantum dots of similar hydrodynamic diameter were injected intracutaneously into five different regions to simultaneously visualize lymphatic flow from the neck and upper trunk of mice. The conjugation of carboxyl groups to quantum dots produced *in vivo* fluorescence images to study the individual and composite drainage patterns towards primary lymph nodes (Figure 15). Such seminal work will aid in the non-invasive prediction of metastasis routes for cancer and has broad implications for predicting cancer metastasis into the lymph nodes.

**Figure 16.**
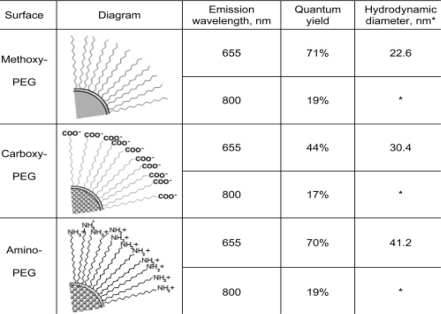
Quantum dot surfaces, measured in 0.01 M sodium borate buffer, pH 8.5. The hydrodynamic diameters are expected to be roughly similar to those of the 655 nm quantum dots. Reprinted with permission from [80].

This review previously described assays using quantum dots to visualize the vasculature associated with cancers, as they have obvious importance to the vitality of the tumor. Taking the reverse approach, Ballou *et al*. mapped sentinel lymph nodes in mice, using carboxyl- and methoxyl- quantum dot injections into tumors themselves (Figure 16) [81]. This unique approach allowed the monitoring of the *in vivo* fluorescence as the quantum dot conjugates moved away from the tumor. Carboxyl-PEG quantum dots were visualized in numerous lymph nodes, whereas methoxyl-PEG quantum dots were only tracked in lymph nodes close to the tumor site. Injecting a mixture of carboxyl and methoxyl quantum dots showed similar migration patterns to the individual injections based on mapping sentinel lymph nodes. Interestingly, both the terminal functional group had little to no effect on the extent of drainage from the tumor into the surrounding lymph nodes and quantum dot size seemed to have little influence on migration patterns.

#### 6.1.5. Circulation/Distribution

A hybrid quantum dot that provided both fluorescence imaging and magnetic resonance imaging was developed [82]. Bakalova *et al*. surrounded quantum dots with paramagnetic particles in a silica sphere to generate a single contrast agent exhibiting both functionalities. The quantum dots provided fluorescent imaging information; the contrast particle provided magnetic resonance imaging (MRI) information. The two techniques were used to simultaneously trace the spheres in blood circulation, providing fluorescent distribution information as well as MRI anatomical reference. Intravenous injection of the spheres did not affect heart rate, blood pressure, or morphology of the blood vessels. Neither the properties of the quantum dots nor the paramagnetic substances were affected by the presence of the other species within the particle. The quantum yield of these hybrid particles was similar to that of the quantum dot particle alone in the silica sphere, and the magnetic properties were unaffected by the presence of the neighboring quantum dots. These hybrid species exhibited low toxicity, and were readily delivered into cultured lung cancer and HeLa cells which were examined by both microscopy and flow cytometry. The two-particle silica spheres could have applications for use in tracing brain vasculature and visualizing neuron, astrocyte, and blood vessel structures.

A number of factors go into the *in vivo* metabolic clearance of quantum dots in mice, including the particle size, coating and coat uniformity, etc. The work of Chen *et al*. employed CdSeS silica-hydroxyl shell particles with a ∼21 nanometer diameter for a tracking and clearance study [83]. The research used ICP-MS to detect cadmium in urine, feces, and other organs in mice to determine the clearance mechanisms of quantum dots *in vivo*. The majority of cadmium was cleared via the urine and feces, at 33.3% and 23.8% respectively. The cadmium concentration peaked in urine 24 – 36 hours post injection and feces 6 – 12 hours post injection. At twelve hours post injection, the cadmium concentration peaked in the liver at 288.1 nanograms per gram of tissue (ng/g tissue), spleen at 158.6 ng/g tissue, lung at 91.0 ng/g tissue, and kidney at 408.7 ng/g tissue. Cadmium remained in the kidney at 120 hours post injection in measurable proportions. In general, excretions of quantum dots that remained unbound were found in the urine, while quantum dots that bound to proteins were found in the feces with bile. Quantum dots that aggregated into larger particles remained in the liver tissue and were difficult to excrete. Continued research that includes different size nanoparticles and different coatings will be extremely important for discussions on long range quantum dot toxicity as well as clearance mechanisms and rates.

Robe *et al*. employed a negatively-charged carboxyl group coating of quantum dots with a hydrodynamic diameter of 16 nanometers to monitor localization and clearance in mice [84]. The negative charge facilitated rapid uptake by the lymph vessels and retention in the lymph nodes. Twenty-four hours post injection, chemical extraction of the axillary lymph nodes, liver, lungs, spleen and kidneys was performed, but only produced detectable fluorescence in the axillary lymph node extraction. Fluorescence spectra of the urine and feces did not produce any visualization of quantum dots and suggested no clearance of the quantum dots in this manner occurred within 24 hours post injection. Fluorescence spectra indicate a majority of the dosage remained at the subcutaneous injection site into the paw of mice and as little as 2–3% of the dosage migrated into the axillary lymph nodes as soon as five minutes post injection. While seemingly contradictory to the above work by Chen *et al*., the experimental differences, such as the use of negatively charged nanoparticles or ICP-MS instrumentation, are enough to warrant more work for both studies, and both works contribute substantially to the science of quantum dots for *in vivo* imaging.

**Figure 17.**
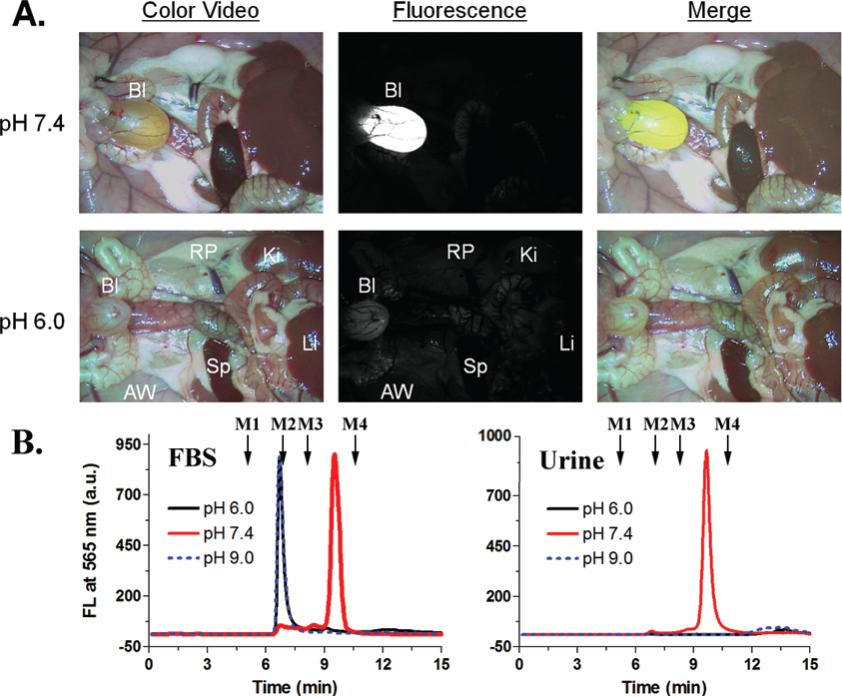
(A) Fluorescence images of rats injected with quantum dot-Cys incubated in FBS at the indicated pH, 4 hours postinjection (Bl, bladder; Ki, kidneys; Li, liver; RP, retroperitoneum; AW, abdominal wall, and Sp, spleen.): color image (left), 565 nm fluorescence (middle), merged (right). (B) GFC analysis of QD-Cys incubated in FBS at various pH (left) and in urine (right) 4 hours postinjection (fluorescence detection at 565 nm). MW markers M1 (thyroglobulin, 670 kDa), M2 (*ç*-globulin, 158 kDa), M3 (ovalbumin, 44 kDa), and M4 (myoglobin, 17 kDa) are shown by arrows. Reprinted with permission from [85].

Lui *et al*. also demonstrated the hydrodynamic size and surface coating of the nanoparticle affected endocytosis and clearance [85]. The authors employed CdSe/ZnCdS quantum dots with a DL-cysteine coating that yielded a six nanometer hydrodynamic diameter nanoparticle. The use of a cysteine coating had an effective neutral charge, which contributed to the lack of aggregation or protein binding in serum. This lack of affinity ensured that these quantum dots could be used without concern of unintended or random interactions. This work also showed that intravenous nanoparticle injection into rats mainly accumulated in the bladder for up to four hours post injection (Figure 17). The authors suspect that the substantially smaller nanoparticle hydrodynamic diameter was below the upper limit for clearance by the renal system. This work highlights another *in vivo* application as cell labels and furthered the understanding bio-distribution and pharmacokinetics. Overall, the research by Robe *et al*., Chen *et al*., and Lui *et al*. are interesting in their assorted approaches, the different permutations of quantum dots employed for their studies, and the varied results they obtained. If anything, these works demonstrate that this field of quantum dot distribution, tracking, and clearance is an area of much research and will be vigorously studies in the future.

### 6.2. Cellular Targeting and Imaging

Quantum dots have utility for a number of types of live cell imaging and detection applications, which can be done externally, but could also require the internalization of quantum dots by cells. As demonstrated in the previous section, internalization of quantum dots for targeting and imaging applications has been achieved using a number of different strategies, including modifications to surface coatings for passive uptake and the use of specific molecules for mediated delivery [39, 82, 86]. These altered surface coatings have been developed to increase the versatility and labeling efficiency of quantum dots for live cells, both prokaryotes and eukaryotes [87–90]. The various surface coatings enhance aqueous solubility, and provide varying functional groups for conjugation to antibodies, peptides, and other biomolecules, and/or reduced nonspecific binding to the cell surface [87,90]. Other improvements to cellular imaging involve variations to the core composition for decreased cytotoxicity and increased biocompatibility [91]. The ability to internalize quantum dot conjugates, along with the increased multiplexing capabilities, offers a major advancement in time- and cost-effectiveness over single-color experiments. Ultimately, these advantages will contribute to the detection of various cell proteins or other components of heterogeneous tumor/tissue samples, including cell mixtures, and will be critical in fields like medical/cancer diagnostics and pathogenic bacteria analysis [92,93].

#### 6.2.1. Labeling/detection

**Figure 18.**
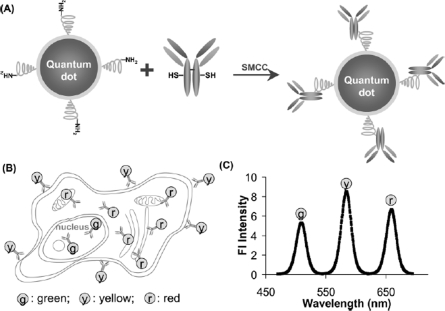
Schematic illustration of bioconjugated quantum dots for multiplexed in situ molecular profiling. (A) Multicolor quantum dot bioconjugates prepared with SMCC activated quantum dots and chemically reduced antibodies. (B) Cell staining using multicolor quantum dot-bioconjugates. (C) Quantification of tumor biomarker expression using wavelength-resolved spectroscopy. Reprinted with permission from [92].

Yezhelyev *et al.* demonstrated the utility of quantum dots for multiplexed cell labeling and analysis by conjugating five different primary antibodies, each labeled with a different color quantum dot, against five cancer biomarkers (HER2, EGFR, PR, ER, and mTOR) [92]. Two human breast cancer cell lines with varying expression levels of the target proteins were formalin-fixed and paraffin-embedded then incubated with quantum dot-antibody conjugates for one hour. Each biomarker was located in a different part of the cell (e.g. cytoplasm, membrane, nucleus), and both cell lines were effectively labeled with all five quantum dot colors (Figure 18). Further analysis of the cells was performed with the essential biomarkers associated with breast cancer detection. The results showed that expression levels of the cancer biomarkers could be quantitatively determined simultaneously, even at very low levels, by measuring the fluorescence intensity of the quantum dots within the cell. Compared to traditional immunohistochemistry assays, quantum dot-immunostaining was more accurate and precise at low protein expression levels. In addition, all quantitative results correlated well with other currently used clinical methods, such as western blotting and fluorescence *in situ* hybridization. This study showed that labeling cells with quantum dots for multiplexed analysis has much potential for use in a clinical setting [92].

Using flow cytometry, quantum dots have been used to detect pathogenic *Escherichia coli* O157:H7 in sample mixtures at very low concentrations [93]. The assay targeted biomolecules on the organism’s cell membrane. Mixtures with increasing ratios of red-labeled innocuous *E. coli* DH5α to (green) quantum dot-labeled pathogenic *E. coli* O157:H7 were analyzed for detection limits. The detection limit results were compared to those of fluorescein-labeled pathogenic *E. coli* at the same concentration, with a full order of magnitude improvement in the detection limit for the quantum dot experiments. The detection limit improvement was further evidenced as cells labeled with fluorescein at ten times the concentration of quantum dots also did not match the results obtained using quantum dots. The detection limit for quantum dot-labeled pathogenic *E. coli* in a background of innocuous *E. coli* was 1% pathogenic cells in 99% innocuous cells, which corresponds to 10^6^ colony forming units per milliliter as the lowest concentration where detection was possible (no distinct peak was observed). The data demonstrated quantum dots are more promising fluorophores for flow cytometry than organic dyes for detection of very low concentrations of cells in a simple mixture. Expanding the experiment by using more complex mixtures would show the complete utility of quantum dots for labeling and detection of bacterial cells.

#### 6.2.2. Cellular Imaging

Bawendi and coworkers recently combined an alloyed ZnCdS shell with heterofunctional, biocompatible, charge-tunable surface coatings for CdSe core quantum dots for use in live cell labeling [87]. The surface coatings consisted of DHLA for coordination to the quantum dot surface, a short PEG spacer for increased solubility and stability, and reactive carboxyl or amino functional groups for conjugation. This heterofunctional scheme, previously demonstrated by Mattoussi *et al*. [44,94–96], applied these quantum dots to demonstrate the combination of an alloyed shell and improved surface coatings for live cell labeling [87]. Quantum dots solubilized with the DHLA/modified-PEG coating had quantum yields from ∼30–40%, were stable over a pH range of ∼5.0–9.5 without affecting brightness, and had a relatively small hydrodynamic diameter (9–12 nanometers), which correlated well with the previous studies from the Mattoussi group that first developed these original surface coatings. The stability over a wide pH range is important for cellular imaging applications because pH varies depending on intracellular location. Also, the smaller size of the quantum dots would allow them to be used for imaging cellular locations that have size restrictions, such as synapses. Both the effective charge and number of reactive functional groups available for conjugation could be controlled while also accounting for solubility. For the live cell labeling experiments, COS7 cells were transfected with human epidermal growth factor receptor (EGFR) and incubated with biotinylated epidermal growth factor (EGF). Specific labeling was achieved with amino-coated quantum dots conjugated to streptavidin, and incubated with the EGF-activated cells. The labeling of the EGF was specific and took place with high affinity. Quantum dot fluorescence did not occur in control cells (those cells not incubated with biotinylated EGF). Further utility of the alloyed shell and surface coating combination was demonstrated with a Fluorescence Resonance Energy Transfer (FRET) assay. While detailed discussions regarding FRET are beyond the scope of this review, many other good reviews exist on the topic [97–100]. Briefly, the FRET assay involved the quantum dots with two functional moieties conjugated to its surface: a covalently conjugated organic dye, and a His_6_-tagged streptavidin through metal-affinity coordination. Including cadmium in the quantum dot shell provided improved metal-affinity driven assembly of the His_6_-tagged proteins [101,102]. The streptavidin allowed specific labeling of biotinylated EGF on the cell surface, resulting in the quantum dots and the organic dye functioning as a FRET pair for visualization. The occurrence of FRET was confirmed by observing the labeled cells at the both quantum dot and dye emission wavelengths. Similar quantum dots with alloyed shells have been fabricated and have exhibited a higher quantum yield than quantum dots with a ZnS shell alone [87,103]. The many advantages of the combination of an alloyed shell and the functionalized PEG surface coating for quantum dots, including charge tunability, improved pH stability, low nonspecific binding, and controlled attachment processes, make this added concept a very useful tool for the future of live cell labeling.

Other techniques to render quantum dots biocompatible for cellular imaging experiments employed denatured proteins on the quantum dot surface. Chemically denatured BSA (dBSA) was used as biocompatible surface coating for CdTe-core quantum dots and provided multiple functional groups available for further conjugation [88]. Quantum dots were coated with dBSA and coupled to anti-*E. coli* antibodies to specifically detect pathogenic *E. coli* O157:H7 in a mixture with *Listeria monocytogenes*. The carboxyl groups of dBSA were available to couple to the antibodies using standard carbodiimide-coupling chemistry. Coating quantum dots with dBSA resulted in a slight decrease in quantum yield.

Phospholipid micelles have been used to encapsulate silicon quantum dots and render them biocompatible [89]. Silicon-core quantum dots are expected to be less toxic than cadmium-containing quantum dots, because of the less toxic core elements. However, silicon quantum dots have found limited use in labeling applications, both *in vitro* and *in vivo,* because aqueous solubilization proves challenging. Micelle coatings are versatile because they can be synthesized to contain a wide range of functional groups, in particular amine and carboxyl groups. Micelle-encapsulated silicon quantum dots retained their desired optical properties and ranged in size from ∼50–120 nanometers. Each micelle contained multiple quantum dots but also exhibited aggregation. Despite aggregation, uptake of micelle-quantum dots was achieved, the extent of which was dependent on the terminal functional groups of the micelle. Live human pancreatic cancer cells were able to internalize amine-functional and transferrin-conjugated micelle-quantum dots, but no uptake of PEG-terminal micelle-quantum dots was observed. While the mechanism is unclear, the functionalization-dependent uptake is most likely due to the overall surface charge of the quantum dot coating [104].

**Figure 19.**
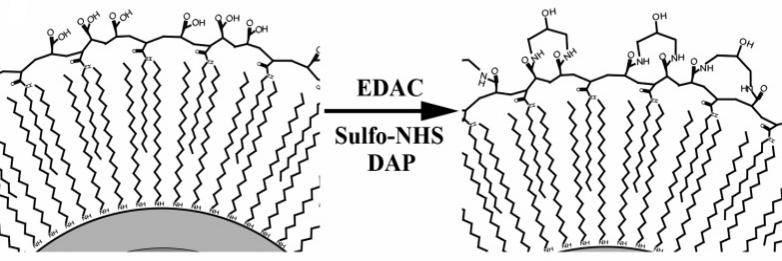
Surface coating chemistry and structure of polymer-encapsulated quantum dots (CdSe/CdS/ZnS). Schematic diagram showing conversion of carboxylated quantum dots (coated with poly(acrylic acid) octylamine) to hydroxylated and cross-linked quantum dots. The small-molecule agent for hydroxylation is 1,3-diamino-2-propanol. Reprinted with permission from [90].

For labeling and imaging of cells, any non-specific binding can be problematic. Nonspecific binding of quantum dots has been observed and is attributed to electrostatic interactions between charged surface ligands and the cell surface [39,62,104,105]. Two independent groups have shown that quantum dots with more neutral surface coatings exhibited lower nonspecific binding to live cells [87,90]. Nie and coworkers developed a hydroxyl-rich surface coating that exhibited almost no nonspecific binding to live cells (Figure 19) [90]. The data indicate a 140-fold decrease in nonspecific binding relative to carboxylated quantum dots and up to 20-fold decrease relative to PEG- and peptide-coated quantum dots. Even at concentrations up to 100 nM, nonspecific binding of quantum dots with hydroxyl coatings to cells was not detected. This hydroxyl rich coating also retained the quantum dot optical properties, had a smaller size relative to those with PEG or polymer coatings (∼13–14 nanometers), and were more stable in acidic buffers than carboxyl-coated quantum dots [39,62,104,105]. In addition, the study done by Bawendi’s group demonstrated a tunable surface charge by using a combination of hydroxyl-containing surface ligands and amino or carboxyl surface ligands [87]. Specifically, as the amount of hydroxyl surface ligands increased, the surface charge decreased concomitant with nonspecific binding to the cell surface. Quantum dots with surface coatings containing a combination of 20% amino and 80% hydroxyl ligands demonstrated the same levels of nonspecific binding as the hydroxyl coating.

As discussed, the metals commonly used to fabricate quantum dots are inherently toxic to living cells, so it would be an important advancement to synthesize quantum dots with inert or biocompatible materials. Silicon bicarbide (SiC) is chemically inert, stable, biocompatible, and has been used to synthesize nanoparticles with cubic symmetry (3*C*-SiC) [91,106–108]. In a recent synthesis, 3*C*-SiC quantum dots with size distribution of ∼0.5–3.5 nanometers and emission wavelengths in the UV-visible region were produced [91]. This seminal work has provided a new material for the fabrication of quantum dots, which did not need an added shell for core protection, and were used directly for live cell imaging. Live fibroblast cells incubated with SiC quantum dots internalized the nanoparticles; a high concentration of quantum dots was found in and near the nucleus while those quantum dots in the cytoplasm were heterogeneously distributed. One week after internalization, no cell death or morphological changes were observed, highlighting the nontoxic nature of SiC quantum dots. While the optical properties are similar to those of their cadmium-containing counterparts, the major advantage of 3*C*-SiC quantum dots is their nontoxic properties and biocompatible core material.

#### 6.2.3. Cellular uptake

Quantum dots can be introduced into cells through a number of different pathways, including transfection, peptide-mediated delivery, or passive uptake (e.g. endocytosis). In a recent study, cellular uptake of quantum dots was studied, where the effects of two different uptake and delivery methods were compared: peptide-mediated delivery and delivery using a transfection reagent [86]. The peptide mediated approach employed the amphiphilic peptide Pep-1 (*KETWWETWWTEW**SQP**KKKRKV**-cystamine*), which is part of the cell-penetrating peptide family that has been previously used for delivery of biomolecules and quantum dots into cells [24,109]. The transfection approach used PolyFect, a transfection reagent that allows for quantum dots to enter the cells through pores created in the cell membrane. The two delivery methods were characterized based on the distribution in the cytoplasm, and eventual redistribution of quantum dots during cell division, as well as the general effect on cell proliferation, surface marker expression, and cell differentiation. Quantum dots delivered by Pep-1 proved better fluorescence markers and were less harmful to cells than quantum dots delivered by using the PolyFect reagent, as Pep-1 delivered quantum dots were more evenly distributed in the cytoplasm, were passed from mother to daughter cells during division, escaped initial lysosome degradation, and did not effect surface marker expression or inhibit cell growth.

While quantum dots can be actively delivered into cells, a number of studies have shown that cells will uptake quantum dots through endocytosis based on their surface coatings. Recently, quantum dots with a paramagnetic probe incorporated in the amine-modified silica shell were used to image a number of different HeLa cell lines [82]. The positive charge of the silica shell allowed for sufficient uptake of quantum dots for fluorescence and magnetic resonance imaging applications. The quantum dots were determined to have entered the cells through endocytosis and distributed in the cytoplasm with a high concentration in the perinuclear region. Identical experiments involving the incubation of cells with quantum dots along with an endocytosis inhibitor resulted in blocked entry into cells, which supports the mechanism of quantum dot uptake was endocytosis. Quantum dots with modified polymer coatings have also demonstrated entry into cells through endocytosis [39]. Polyethylenimine (PEI) contains a large network of amine groups and has a positive charge that facilitates endocytosis. While PEI-coated quantum dots move easily across the cell membrane, they are lethal to cells. Grafting the PEI coating with PEG reduces the harmful effects without affecting uptake, but the extent of grafting influences intracellular distribution. Quantum dots with a surface coating containing four PEG grafts per PEI were trapped in organelles around the nucleus while those with a surface coating containing only two PEG grafts per PEI were released from organelles and distributed in the cytoplasm. These experiments demonstrate surface coatings are important for both cellular uptake and intracellular distribution, both of which are critical for applications such as drug- and small molecule delivery.

### 6.3. Other Applications

Beside *in vivo* and cellular imaging, quantum dots can be used for a wide range of other applications relevant to the life sciences, such as fixed tissue analysis, spectral encoding of microparticles, and even unique applications such as quantitative analysis of ions. While each of these topics may be less conventional than *in vivo* analysis, they demonstrate the versatility of quantum dots and each has relevance to biological applications. Analysis of fixed tissue samples is a standard technique that is important in cancer diagnostics and morphology studies [92,110]. Encoding microparticles with quantum dots effectively increases the multiplexing capabilities of a system, useful in high-throughput or multiplexed detection of biomolecules or biomolecular interactions [57,111]. Finally, quantum dots have been used for quantitative determination of metal ions in complex samples or glucose in clinical serum samples [112,113].

#### 6.3.1. Fixed tissue analysis

Quantum dot conjugates for detection of cancer biomarkers in cultured human breast cancer cells was described previously in the applications section [92]. The same researchers also used the same quantum dot-antibody conjugates for simultaneous detection and quantitative analysis of cancer markers in clinical tissue samples. The brightness and photostability of quantum dots are advantageous for this type of analysis because they are much brighter than any background autofluorescence from the tissue samples. In addition, the background autofluorescence can be photobleached with prolonged exposure to excitation light, while quantum dots remain unaffected. Comparing the results from the study to known immunohistochemistry data for three markers, the cancer marker expression levels showed little or no deviation from the known data. While the results are promising, the authors suggest a number of improvements to the method, including a more compact quantum dot probe for deeper tissue penetration, improved conjugation protocols to control the number and orientation of antibodies on the quantum dot surface, and the inclusion of standardization markers.

In a similar study, quantum dots were conjugated to antibodies and used to probe multiple targets with differing cellular locations (surface, cytoplasm, and nuclear) in clinical tissue samples [110]. Five different quantum dot-antibody conjugates and one organic fluorophore-antibody conjugate (4,6-diamidino-2-phenylindole, DAPI), were used to simultaneously detect the location of five antigens specific to five different cell types to gather information about the immunological microanatomy of human lymphoid tissue. This research showed that streptavidin-conjugated quantum dots were more effective than direct conjugation of the antibodies to the quantum dot surface. Tissue samples were sequentially stained with quantum dot-antibody conjugates and the resulting imaging was analyzed using an emission fingerprinting technique [114]. The images could be overlaid or separated into constituent quantum dot colors based on channels in the imaging system. By analyzing the individual images, location of each antigen and the corresponding cell types could be determined and compared to results of other morphology-determining techniques.

#### 6.3.2. Optical encoding

**Figure 20.**
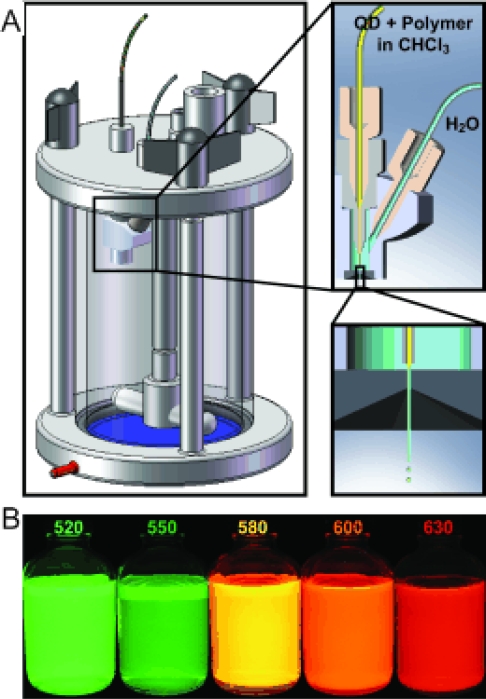
CCFF barcodes. (A) The CCFF process includes the concentration reactor and the production nozzle. An enlargement is shown in the top right with a cross-sectional diagram of the flow-focusing nozzle in which a quantum dot fluorophore solution in chloroform with 4% dissolved polymer is introduced through the top (yellow). The flow-focusing fluid (deionized water) is introduced from the right (blue). A close-up view of the quantum-dot–polymer solution being focused and “pinched off ” into microscopic droplets by the water flow is shown on the bottom right corner. Each small droplet formed is an active polymer microbead with homogeneous quantum dot encoding. (B) UV illuminated picture of the five stock solutions of quantum dots used to generate a 105-barcode library. The number printed on each bottle represents the fluorescence emission wavelength in nanometers of the respective quantum dots. Reprinted with permission from [57].

A novel method for mass preparation of quantum dot barcodes was recently developed, enabling a vast number of differentiable microspheres, each impregnated with a series of quantum dots that provided a unique emission spectra pattern that was employed as barcodes [57]. The system, called concentration-controlled flow-focusing (CCFF), contains a pressurized vessel and a nozzle inlet system (Figure 20). The pressurized vessel allows real-time control over changes in total volume and solution concentrations. The nozzle system controls the flow of reagents to produce identical microspheres. The reproducibility of each microsphere is critical to controlling the barcode. Two inlets in the nozzle combine the aqueous and the organic phases, comprised of chloroform, quantum dots, and a polymer. As the two solutions mix, the polymer solidifies around the quantum dots creating a homogeneously dispersed microsphere. The polymer, such as poly(styrene-*co*-maleic anhydride), also provides stability, because after the microsphere shell is formed, the free anhydride functional groups are hydrolyzed in water. Because the barcodes are produced in a single step, directly in an aqueous solution, and no purification steps are necessary post-synthesis.

**Figure 21.**
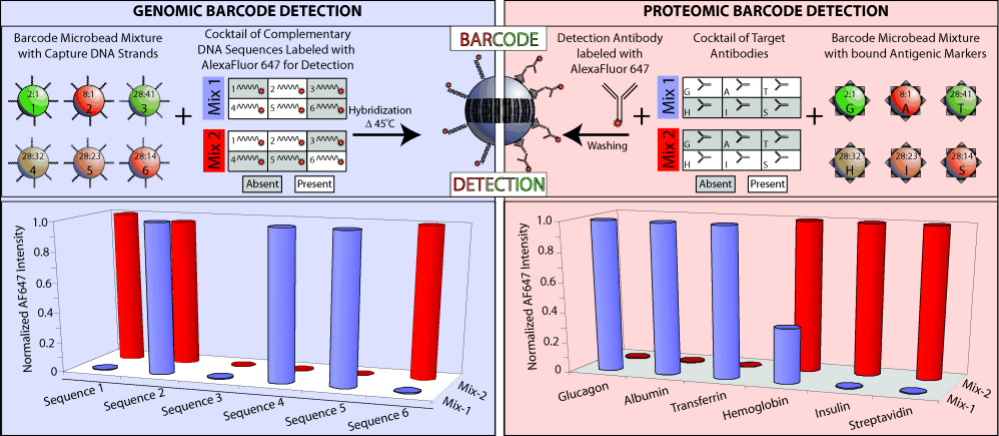
Multiplexed protein and DNA assays. Six DNA and six proteomic multiplexed assays were performed on the QD-barcoded beads. Triplicates were performed for each experiment and computed standard deviation was less than 10% in all cases. Reprinted with permission from [57].

Using varying concentrations and ratios of five CdSe/ZnS quantum dots, a library of more than 100 different barcoded microspheres was produced, each with its own unique spectral “fingerprint” (Figure 21). Other methods currently used for optical encoding using quantum dots, including the ‘swelling’ technique [55] and incorporating quantum dots into a polymer coating have experienced stability issues [115]. The described synthesis method solves most stability problems; the quantum dot barcodes are stable over a wide pH range, at high temperatures (up to 95°C), with no appreciable leaking of quantum dots from the polymer shell. The barcodes in the multiplexed analysis experiment employed no more than three types of quantum dots per microsphere, with the relative quantum dot ratios limited to 1x, 2x, and 4x. Six barcoded microspheres were separately conjugated to six different single-stranded DNA probes for multiplexed detection of DNA targets labeled with an organic dye. The barcode microspheres were added to target solutions with the barcodes and hybridization efficiency monitored using flow cytometry. The DNA-conjugated quantum dot barcoded microspheres specifically detected their complementary target DNA sequences from complex mixtures (Figure 21). The experiment exhibited little or no instance of cross-reactivity or nonspecific binding. The same procedure was carried out using six antibody-conjugated barcoded microspheres and the corresponding dye-labeled antigens to the same results. A limitation of the method is the size of the microspheres, in the range of 4–20 micron, as this relatively large size has the potential to be a hindrance in biological systems.

**Figure 22.**
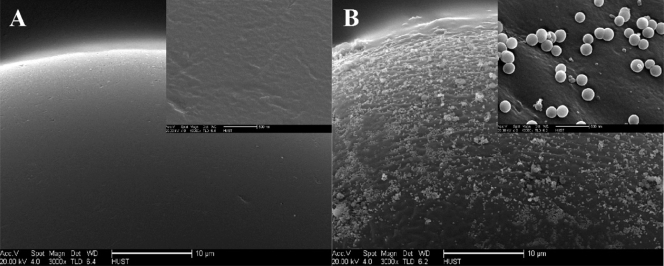
Scanning electron micrograph images of the beads’ surfaces: original polystyrene beads (A) and the Si quantum dots coated beads (B) The insets are magnified 50,000 times, and the images were taken under the same conditions. Reprinted with permission from [111].

Instead of synthesizing quantum dot barcoded spheres, polystyrene microbeads can be coated with quantum dots for use as optically encoded fluorescent markers (Figure 22) [111]. Carboxyl-functionalized polystyrene microbeads were coated with silica encapsulated-CdSe/ZnS water solubilized quantum dots and used for antibody-antigen studies. Encapsulating the water-soluble quantum dots in silica did not affect their optical properties, but increased their photostability under prolonged irradiation. Encapsulating the quantum dots in silica proved critical for rendering the microspheres optically active, as functionalized beads prepared with only water-soluble quantum dots did not fluoresce. Encapsulating quantum dots in a silica shell nearly eliminated ‘leaking’ of quantum dots off the beads, which is a common problem when coating microbeads with quantum dots [116, 117]. The silica-quantum dot-coated functionalized microspheres were covalently linked to human IgG and were shown to selectively detect fluorescein-labeled anti-human IgG and not interact with other non-specific IgG solutions. A number of methods are available to employ microsphere coded with quantum dots for biological analysis, and quantum dots are ideal fluorophores for optical encoding because of their bright, narrow and controllable optical properties.

#### 6.3.3. Quantitative determination

In a truly unique application, quantum dots have been used in sensing applications for quantitative determination of ions due to certain species possessing the unique ability to quench quantum dot fluorescence. Recently, Zhang *et al*. used l-cysteine coated CdSe/CdS quantum dots for copper(II) determination in various vegetable samples [112]. Other quantum dot core/shell and solubilization ligand combinations have previously been used for copper(II) determination, but with only limited quantitative applicability [40,118,119]. The l-cysteine coating was applied to the core/shell nanoparticles by ligand exchange, and provided an increased robustness; the quantum dots were soluble over a pH range of 2–10 and stable in aqueous solution for up to five days if kept in the dark. The presence of free cysteine increased the stability of the system, but the quantum dot fluorescence intensity decreased, resulting in a decreased dynamic range of detectable copper ion concentrations. The sensitivity of quantum dot fluorescence to Cu^2+^ ions was shown by exposing quantum dots to a number of ions and measuring the fluorescence of the mixture. A large number of metal and halogen ions at a concentrations at or below ∼2 μM were probed, including Fe^3+^, Mn^2−^, Ni^2+^, Cd^2+^, and Co^2+^ ions, among others, none of which influenced quantum dot emissions. In contrast, quantum dot emission was quenched by almost 9% when exposed to 10 nM Cu^2+^ ions. The sensitivity of quantum dots for copper determination was measured in the presence of these other ions in solution. In 10 nM Cu^2+^ solutions, the lowest concentration of any ion to affect fluorescence was between 2 μM and 50 μM (a minimum 200 times greater than the copper concentration), and the dynamic range of detectable Cu^2+^ was 10–200 nM with a detection limit was 3 nM. The relationship between the amount of Cu^2+^ in solution and the decrease in quantum dot fluorescence was linear, and allowed simple determination of the amount of copper(II) in vegetable samples. Analysis of five vegetables samples with the l-cysteine-coated quantum dot sensors generated results that correlated well with the results obtained from inductively coupled plasma-optical emission spectroscopy. The unique ability of certain ions to quench quantum dot fluorescence lends itself well for the application of quantum dots as nanosensors for quantitative determination of ions. Compared to other methods, coated quantum dots with l-cysteine exhibited a wider dynamic range and a lower detection limit [40,118,119].

As the previous section demonstrated quantum dot emissions can be quenched, a logical expectation would be that quantum dots could also participate in FRET assays. In such cases, quantum dots conjugated to species such as biomolecules and other nanoparticles would function as FRET-based nanosensors. A recent study employed a novel assembly of nanoparticles and biomolecules for sensitive, selective quantitative determination of glucose in serum [113]. The assay was based on the binding interactions of concanavalin A (ConA), a lectin that binds certain sugars with high affinity. The sensing scheme involved ConA-conjugated quantum dots and gold nanoparticles conjugated to β-cyclodextrins (β-CDs). In glucose-free solution, ConA bound to the β-CDs, and the fluorescence of the quantum dot was quenched due to the proximity of the gold nanoparticle. The full FRET scheme had the quantum dot act as the FRET donor and the gold nanoparticle as the FRET acceptor. Glucose competitively binds to the active sites on ConA, which inhibits or displaces the β-CDs-gold nanoparticle conjugate. The release of the gold nanoparticle conjugate “turns off” the FRET quenching and restores the quantum dot emission. Experiments were performed to correlate the fluorescence and concentration of glucose, and the detection limit for glucose was determined to be 50 nM. In addition, the results of glucose determination were not affected by a number of coexisting biological molecules or other ions at relevant concentrations. The sensitivity and selectivity demonstrated by the enabled its application for glucose detection in human serum samples. In human serum samples, the amount of glucose was determined using the quantum dot-ConA-β-CDs-gold nanoparticle system, and the results compared well with a common certified method. The combined selectivity, sensitivity, and FRET efficiency allowed for quantitative determination of glucose levels in clinical samples and the small size of the sensor system has the potential for use in direct determination of glucose levels in single cells or bacterial cultures.

## 7. Other Nanoparticles

Other nanoparticles, not comprised of semiconductor materials, have also been applied to some of same applications as quantum dots. While this section will not specifically detail work performed with quantum dots, in vivo applications involving nanoparticles is moving in a new direction, in large part, due to the successes associated with quantum dots. This next section will briefly highlight seminal work involving other nanoparticles, including gold nanoparticles, fluorescent dye-doped silica nanoparticles, Raman-active dye-embedded nanoparticles, and nanoworms [120–124]. Much like quantum dots, these nanoparticles have been conjugated to antibodies, DNA, or other biomolecules for use as *in vivo* imaging probes. These various reporter species, similar to quantum dots, are proving to be advantageous with general applications such as immunoassays, multiplexed analyses, and ultrasensitive detection schemes [31,125–127]. Although not quantum dots by definition, these novel reporter moieties have comparable attributes and are being put forth towards novel applications, some of which will be briefly discussed below.

### 7.1. Fluorescence measurements: gold and silica nanoparticles

Like quantum dots, gold nanoparticles and fluorescent-dye doped silica nanoparticles can be employed for optical measurements and exhibit size-tunable properties [120,128,129]. The utility of gold nanoparticles conjugated to DNA is especially pronounced, as these conjugates exhibit improved characteristics as compared to the free biological species, such as increased stability at elevated temperatures [128], enhanced binding properties [121,130], and sharper melting transitions [131]. Increased nanoparticle-conjugate stability is critical for performing DNA hybridization and denaturization experiments. While the nature of the biomolecule bestows the recognition specificity, the increased stability and sharp melting transition that have been demonstrated with gold nanoparticle-oligonucleotide conjugates provides enhanced selectivity. When probe and target sequences are conjugated to separate gold nanoparticles and hybridized, the sharp transition in melting temperature stems from the fact that an extended network of hybridized nanoparticle-oligonucleotides conjugates is created [131]. The network acts collectively, and remains uninterrupted if a single hybridization pair dissociates; a specific, higher temperature is required to completely denature the entire network, leading to a sharper melting transition. These conjugates have exhibited sharper melting transitions, which allow for distinction between sequences with as few as a single base pair mismatch [121,130,131]. Gold nanoparticle bioconjugates have been used in ultrasensitive *in vivo* DNA, protein, and abnormal cell detection [121,123].

Unlike gold nanoparticles, silica particles themselves are not optically active without modification. However, silica nanoparticles have been ‘doped’ with different dyes to render them applicable in biological imaging [120,124]. Fluorescent dyes can be incorporated into the silica during synthesis or can be attached to the particle surface after synthesis, imparting them with luminescent properties. The resulting dye-doped silica nanoparticles are more photostable than the free dye [126], which makes them useful for *in vivo* applications, such as imaging and targeting [120,124].

### 7.2. Raman scattering measurements: dye-embedded nanoparticles

Raman spectroscopy has been utilized as an alternative to fluorescence analysis of nanoparticles. The many advantages of Raman spectroscopy can be significant, including the ability to excite the reporters at any wavelength, limited photobleaching, and the wide variety of Raman tags that exist [132]. With Raman reporters each having a unique, fingerprint-like spectrum, potentially hundreds of different Raman tags are available [133]. In addition, surface-enhanced Raman scattering has an enormous signal enhancement factor, determined to be 10^14^–10^15^ fold [134–137]. While the enhancement mechanism is unclear, the effect is well documented, and as such, Raman reporters have the potential to be much more sensitive than typical fluorescent reporters [31,127].

To render typical core/shell nanoparticles Raman-active, two major strategies have been developed: attachment of the reporter to the nanoparticle shell and embedding the reporter within the shell [125]. Embedding the Raman reporter internally has shown to be preferable because the reporter is protected from the environment [132], and does not contribute to toxicity. The most common Raman reporters embedded in nanoparticle shells are Raman-active organic dyes, and a number of methods have been employed to embed them in nanoparticles for use in biological applications [31,125,127], Dye-embedded nanoparticles are designed much like quantum dots, with a metallic core for optical enhancement (e.g. gold) and a shell for protection and conjugation (e.g. silica). Conjugating a peptide or oligonucleotide to the silica shell renders the nanoparticle useful in a number of life science applications, including *in vivo* imaging.

### 7.3. Magnetic resonance measurements: nanoworms

**Figure 23.**
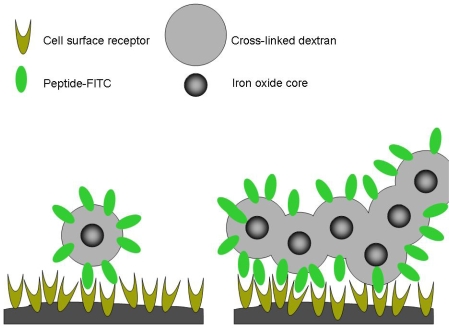
Internalization of nanoworms and nanospheres conjugated with peptides into cells. A conceptual scheme illustrating the increased multivalent interactions expected between receptors on a cell surface and targeting ligands on a nanoworm compared with a nanosphere. Adapted with permission from [122].

Magnetic resonance imaging (MRI) has also employed nanoparticles for *in vivo* imaging. More specifically, a different type of nanoparticle, termed nanoworm, has recently been developed for *in vivo* tumor targeting and imaging [122]. Nanoworms, as their name suggests have longer dimensions, but also a different composition than quantum dots. This work by Sailor *et al*. employ elongated, dextran-coated particles composed of a linear aggregate of 5–10 iron oxide cores and a total length between ∼50–80 nm. The elongated nanoworm structure allowed a larger number of interactions between peptides conjugated to the nanoworm and cell-surface receptors to take place relative to nanospheres of similar composition (Figure 23). For example, nanoworms were compared to other nanoparticles in bloodstream circulation experiments, which is a crucial aspect of effective *in vivo* imaging [138,139]. The elongated structure of nanoworms did not affect the amount of time in the bloodstream compared to traditional circular nanoparticles. While circulation times were similar, the distribution of the nanoparticles differed; the ratio of nanoworms in the kidney relative to the liver was lower than nanosphere particles, but the ratio of particles in the spleen to the liver was higher for nanoworms [122]. This concept is essentially structure-function based, with more peptides capable of interacting with cell surface-receptors, without sacrificing residence time in the bloodstream. This facet of enhanced interaction would focus the efficiency of peptide-modified nanoworms towards tumors [122]. Also, the extended length provided an increased magnetic relativity, resulting in a higher magnetic resonance contrast. The higher magnetic contrast allowed for a wider range of concentrations to be used in a single experiment. In terms of *in vivo* imaging, passive accumulation of nanoworms in tumors has demonstrated increases relative to nanospheres, and once nanoworms enter tumors, they exhibit a high residence time and do not readily re-enter the bloodstream [122]. The confinement of nanoworms in tumors and the larger relative number of peptide-receptor interactions will allow more effective and sensitive imaging of tumor tissue using magnetic resonance imaging.

## 8. Summary

Quantum dots are now becoming commonplace for *in vivo* imaging. While their use is still behind that of traditional or conventional reporters, their frequency of use is dramatically increasing. This dramatic increase is in part, due to the numerous advantageous properties exhibited by quantum dots, including a robust signal strength, increased ability to multiplex, resistance to photobleaching, and the customizable nature of their fabrication and conjugation protocols. As research, such as the seminal work outlined in this review continue, the revelation as to what quantum dots can do for applications such as *in vivo* imaging and analysis, as well as the diversity with which quantum dots can be applied, will open new avenues of research and continue the development of methods, instrumentation, and diagnostics that will truly revolutionize the life sciences.

## Figures and Tables

**Table 1.** Core Compositions, Size Ranges, and Emission Wavelength Ranges of Quantum Dots.

*Core Composition*	*Size Range (nm)*	*Emission Range, reference*
ZnSe	∼4.3–6.0	UV, Visible (Size dependent), [7,8]
ZnSe:Mn	∼2.7∼6.3	UV, Visible (Size dependent), [9]
CdSe	∼1.0 up to 25	Visible, [1,3,10–14]
CdS	∼1.0–6.0	UV, Visible (Size dependent), [3,8,15]
CdTe	∼2.0–8.0	Visible, [3,15]
InP	3.0–4.6	UV, Visible, Near IR (Size dependent) [1,16]
	∼2.6–4.6	
InAs	2.8–6.0	IR, [1]
GaP	∼2.0–3.0	UV, Visible (Size dependent), [16]
GaInP_2_	∼2.5–6.5	UV, Visible (Size dependent), [16]
PbSe	3–8 (small), 8–12 (large)	Near/mid-IR (Size dependent), [17–19]
SnTe	4.5–15	Mid-IR, [20]

## References

[1] Bruchez M, Moronne M, Gin P, Weiss S, Alivisatos AP (1998). Semiconductor Nanocrystals as Fluorescent Biological Labels. Science.

[2] Chan WCW, Nie S (1998). Quantum Dot Bioconjugates for Ultrasensitive Nonisotopic Detection. Science.

[3] Murray CB, Norris DJ, Bawendi MG (1993). Synthesis and Characterization of Nearly Monodisperse CdE (E = sulfur, selenium, tellurium) Semiconductor Nanocrystallites. J. Am. Chem. Soc.

[4] Hines MA, Guyot-Sionnest P (1996). Synthesis and Characterization of Strongly Luminescing ZnS-Capped CdSe Nanocrystals. J. Phys. Chem.

[5] Danek M, Jensen KF, Murray CB, Bawendi MG (1996). Synthesis of Luminescent Thin-Film CdSe/ZnSe Quantum Dot Composites Using CdSe Quantum Dots Passivated with an Overlayer of ZnSe. Chem. Mater.

[6] Dabbousi BO, Rodriguez-Viejo J, Mikulec FV, Heine JR, Mattoussi H, Ober R, Jensen KF, Bawendi MG (1997). (CdSe)ZnS Core-Shell Quantum Dots: Synthesis and Characterization of a Size Series of Highly Luminescent Nanocrystallites. J. Phys. Chem. B.

[7] Hines MA, Guyot-Sionnest P (1998). Bright UV-Blue Luminescent Colloidal ZnSe Nanocrystals. J. Phys. Chem. B.

[8] Yu WW, Peng X (2002). Formation of High-Quality CdS and Other II–VI Semiconductor Nanocrystals in Noncoordinating Solvents: Tunable Reactivity of Monomers. Angew. Chemie.

[9] Norris DJ, Yao N, Charnock FT, Kennedy TA (2001). High-Quality Manganese-Doped ZnSe Nanocrystals. Nano Lett.

[10] Kuno M, Lee JK (1997). The Band Edge Luminescence of Surface Modified CdSe Nanocrystallites: Probing the Luminescing State. J. Chem. Phys.

[11] Talapin DV, Rogach AL, Kornowski A, Haase M, Weller H (2001). Highly Luminescent Monodisperse CdSe and CdSe/ZnS Nanocrystals Synthesized in a Hexadecylamine-Trioctylphosphine Oxide-Trioctylphospine Mixture. Nano Lett.

[12] Qu L, Peng ZA, Peng X (2001). Alternative Routes Toward High Quality CdSe Nanocrystals. Nano Lett.

[13] Katari JEB, Colvin VL, Alivisatos AP (1994). X-ray Photoelectron Spectroscopy of CdSe Nanocrystals with Applications to Studies of the Nanocrystal Surface. J. Phys. Chem.

[14] Krueger KM, Al-Somali AM, Falkner JC, Colvin VL (2005). Characterization of Nanocrystalline CdSe by Size Exclusion Chromatography. Anal. Chem.

[15] Peng ZA, Peng X (2001). Formation of High-Quality CdTe, CdSe, and CdS Nanocrystals Using CdO as Precursor. J. Am. Chem. Soc.

[16] Micic OI, Sprague JR, Curtis CJ, Jones KM, Machol JL, Nozik AJ, Giessen H, Fluegel B, Mohs G, Peyghambarian N (1995). Synthesis and Characterization of InP, GaP, and GaInP2 Quantum Dots. J. Phys. Chem.

[17] Murray CB, Sun S, Gaschler W, Doyle H, Betley TA, Kagan CR (2001). Colloidal Synthesis of Nanocrystals and Nanocrystal Superlattices. IBM J. Res. Dev.

[18] Du H, Chen C, Krishnan R, Krauss TD, Harbold JM, Wise FW, Thomas MG, Silcox J (2002). Optical Properties of Colloidal PbSe Nanocrystals. Nano Lett.

[19] Pietryga JM, Schaller RD, Werder D, Stewart MH, Klimov VI, Hollingsworth JA (2004). Pushing the Band Gap Envelope: Mid-Infrared Emitting Colloidal PbSe Quantum Dots. J. Am. Chem. Soc.

[20] Kovalenko MV, Heiss W, Shevchenko EV, Lee J-S, Schwinghammer H, Alivisatos AP, Talapin DV (2007). SnTe Nanocrystals: A New Example of Narrow-Gap Semiconductor Quantum Dots. J. Am. Chem. Soc.

[21] Weller H (1993). Colloidal Semiconductor Q-Particles: Chemistry in the Transition Region Between Solid State and Molecules. Angew. Chemie.

[22] Peng X, Schlamp MC, Kadavanich AV, Alivisatos AP (1997). Epitaxial Growth of Highly Luminescent CdSe/CdS Core/Shell Nanocrystals with Photostability and Electronic Accessibility. J. Am. Chem. Soc.

[23] Gao X, Nie S (2004). Quantum Dot-Encoded Mesoporous Beads with High Brightness and Uniformity. Anal. Chem.

[24] Mattheakis LC, Dias JM, Choi Y-J, Gong J, Bruchez MP, Liu J, Wang E (2004). Optical Coding of Mammalian Cells Using Semiconductor Quantum Dots. Anal. Biochem.

[25] Jaiswal JK, Mattoussi H, Mauro JM, Simon SM (2003). Long-term Multiple Color Imaging of Live Cells Using Quantum Dot Bioconjugates. Nat. Biotechnol.

[26] Chen F, Gerion D (2004). Fluorescent CdSe/ZnS Nanocrystal-Peptide Conjugates for Long-term, Nontoxic Imaging and Nuclear Targeting in Living Cells. Nano Lett.

[27] Derfus AM, Chan WCW, Bhatia SN (2004). Probing the Cytotoxicity of Semiconductor Quantum Dots. Nano Lett.

[28] Hoshino A, Fujioka K, Oku T, Suga M, Sasaki YF, Ohta T, Yasuhara M, Suzuki K, Yamamoto K (2004). Physicochemical Properties and Cellular Toxicity of Nanocrystal Quantum Dots Depend on Their Surface Modification. Nano Lett.

[29] Kirchner C, Liedl T, Kudera S, Pellegrino T, MunozJavier A, Gaub HE, Stolzle S, Fertig N, Parak WJ (2005). Cytotoxicity of Colloidal CdSe and CdSe/ZnS Nanoparticles. Nano Lett.

[30] Ryman-Rasmussen JP, Riviere JE, Monteiro-Riviere NA (2006). Penetration of Intact Skin by Quantum Dots with Diverse Physicochemical Properties. Toxicol. Sci.

[31] Zhang T, Stilwell JL, Gerion D, Ding L, Elboudwarej O, Cooke PA, Gray JW, Alivisatos AP, Chen FF (2006). Cellular Effect of High Doses of Silica-Coated Quantum Dot Profiled with High Throughput Gene Expression Analysis and High Content Cellomics Measurements. Nano Lett.

[32] Dubertret B, Skourides P, Norris DJ, Noireaux V, Brivanlou AH, Libchaber A (2002). *In Vivo* Imaging of Quantum Dots Encapsulated in Phospholipid Micelles. Science.

[33] Hoshino A, Hanaki K, Suzuki K, Yamamoto K (2004). Applications of T-lymphoma Labeled with Fluorescent Quantum Dots to Cell Tracing Markers in Mouse Body. Biochem. Biophys. Res. Commun.

[34] Larson DR, Zipfel WR, Williams RM, Clark SW, Bruchez MP, Wise FW, Webb WW (2003). Water-Soluble Quantum Dots for Multiphoton Fluorescence Imaging *In Vivo*. Science.

[35] Wu X, Liu H, Liu J, Haley KN, Treadway JA, Larson JP, Ge N, Peale F, Bruchez MP (2003). Immunofluorescent Labeling of Cancer Marker Her2 and Other Cellular Targets with Semiconductor Quantum Dots. Nat. Biotechnol.

[36] Kim S, Lim YT, Soltesz EG, De Grand AM, Lee J, Nakayama A, Parker JA, Mihaljevic T, Laurence RG, Dor DM (2004). Near-infrared Fluorescent Type II Quantum Dots for Sentinel Lymph Node Mapping. Nat. Biotechnol.

[37] Medintz IL, Konnert JH, Clapp AR, Stanish I, Twigg ME, Mattoussi H, Mauro JM, Deschamps JR (2004). A Fluorescence Resonance Energy Transfer-derived Structure of a Quantum Dot-protein Bioconjugate Nanoassembly. Proc. Natl. Acad. Sci. USA.

[38] Ryman-Rasmussen JP, Riviere JE, Monteiro-Riviere NA (2006). Surface Coatings Determine Cytotoxicity and Irritation Potential of Quantum Dot Nanoparticles in Epidermal Keratinocytes. J. Invest. Dermatol.

[39] Duan H, Nie S (2007). Cell-Penetrating Quantum Dots Based on Multivalent and Endosome-Disrupting Surface Coatings. J. Am. Chem. Soc.

[40] Chen Y, Rosenzweig Z (2002). Luminescent CdS Quantum Dots as Selective Ion Probes. Anal. Chem.

[41] Cui B, Wu C, Chen L, Ramirez A, Bearer EL, Li W-P, Mobley WC, Chu S (2007). One at a Time, Live Tracking of NGF Axonal Transport Using Quantum Dots. Proc. Natl. Acad. Sci. USA.

[42] Nirmal M, Dabbousi BO, Bawendi MG, Macklin JJ, Trautman JK, Harris TD, Brus LE (1996). Fluorescence Intermittency in Single Cadmium Selenide Nanocrystals. Nature.

[43] Chen Y, Vela J, Htoon H, Casson JL, Werder DJ, Bussian DA, Klimov VI, Hollingsworth JA (2008). Multishell CdSe Nanocrystal Quantum Dots with Suppressed Blinking. J. Am. Chem. Soc.

[44] Mattoussi H, Mauro JM, Goldman ER, Anderson GP, Sundar VC, Mikulec FV, Bawendi MG (2000). Self-Assembly of CdSe-ZnS Quantum Dot Bioconjugates Using an Engineered Recombinant Protein. J. Am. Chem. Soc.

[45] Akerman ME, Chan WCW, Laakkonen P, Bhatia SN, Ruoslahti E (2002). Nanocrystal Targeting *In Vivo*. Proc. Natl. Acad. Sci. USA.

[46] Gussin HA, Tomlinson ID, Little DM, Warnement MR, Qian H, Rosenthal SJ, Pepperberg DR (2006). Binding of Muscimol-Conjugated Quantum Dots to GABA Receptors. J. Am. Chem. Soc.

[47] Gao X, Chan WCW, Nie S (2002). Quantum-dot Nanocrystals for Ultrasensitive Biological Labeling and Multicolor Optical Encoding. J. Biomed. Opt.

[48] Smith A, Ruan G, Rhyner M, Nie S (2006). Engineering Luminescent Quantum Dots for *In Vivo* Molecular and Cellular Imaging. Ann. Biomed. Eng.

[49] Parak WJ, Boudreau R, Le Gros M, Gerion D, Zanchet D, Micheel CM, Williams SC, Alivisatos AP, Larabell C (2002). Cell Motility and Metastatic Potential Studies Based on Quantum Dot Imaging of Phagokinetic Tracks. Adv. Mater.

[50] Kloepfer JA, Mielke RE, Wong MS, Nealson KH, Stucky G, Nadeau JL (2003). Quantum Dots as Strain- and Metabolism-Specific Microbiological Labels. Appl. Environ. Microbiol.

[51] Pinaud F, King D, Moore H-P, Weiss S (2004). Bioactivation and Cell Targeting of Semiconductor CdSe/ZnS Nanocrystals with Phytochelatin-Related Peptides. J. Am. Chem. Soc.

[52] Sukhanova A, Devy J, Venteo L, Kaplan H, Artemyev M, Oleinikov V, Klinov D, Pluot M, Cohen JHM, Nabiev I (2004). Biocompatible Fluorescent Nanocrystals for Immunolabeling of Membrane Proteins and Cells. Anal. Biochem.

[53] Zhang CY, Johnson LW (2007). Quantifying RNA-Peptide Interaction by Single-quantum Dot-Based Nanosensor: An Approach for Drug Screening. Anal. Chem.

[54] Goldman ER, Clapp AR, Anderson GP, Uyeda HT, Mauro JM, Medintz IL, Mattoussi H (2004). Multiplexed Toxin Analysis Using Four Colors of Quantum Dot Fluororeagents. Anal. Chem.

[55] Han M, Gao X, Su JZ, Nie S (2001). Quantum-dot-tagged Microbeads for Multiplexed Optical Coding of Biomolecules. Nat. Biotechnol.

[56] Gerion D, Parak WJ, Williams SC, Zanchet D, Micheel CM, Alivisatos AP (2002). Sorting Fluorescent Nanocrystals with DNA. J. Am. Chem. Soc.

[57] Fournier-Bidoz S, Jennings TL, Klostranec JM, Fung W, Rhee A, Li D, Chan WCW (2008). Facile and Rapid One-Step Mass Preparation of Quantum-Dot Barcodes. Angew Chemie.

[58] Voura EB, Jaiswal JK, Mattoussi H, Simon SM (2004). Tracking Metastatic Tumor Cell Extravasation with Quantum Dot Nanocrystals and Fluorescence Emission-Scanning Microscopy. Nature Med.

[59] Dabbousi BO, Murray CB, Rubner MF, Bawendi MG (1994). Langmuir-Blodgett Manipulation of Size-Selected CdSe Nanocrystallites. Chem. Mater.

[60] Danek M, Jensen KF, Murray CB, Bawendi MG (1994). Electrospray Organometallic Chemical Vapor Deposition—A Novel Technique for Preparation of II–VI Quantum Dot Composites. Appl. Phys. Lett.

[61] Michalet X, Pinaud FF, Bentolila LA, Tsay JM, Doose S, Li JJ, Sundaresan G, Wu AM, Gambhir SS, Weiss S (2005). Quantum Dots for Live Cells, *In Vivo* Imaging, and Diagnostics. Science.

[62] Gerion D, Pinaud F, Williams SC, Parak WJ, Zanchet D, Weiss S, Alivisatos AP (2001). Synthesis and Properties of Biocompatible Water-Soluble Silica-Coated CdSe/ZnS Semiconductor Quantum Dots. J. Phys. Chem. B.

[63] Chen CC, Yet CP, Wang HN, Chao CY (1999). Self-Assembly of Monolayers of Cadmium Selenide Nanocrystals with Dual Color Emission. Langmuir.

[64] Mitchell GP, Mirkin CA, Letsinger RL (1999). Programmed Assembly of DNA Functionalized Quantum Dots. J. Am. Chem. Soc.

[65] Mattoussi H, Mauro JM, Goldman ER, Green TM, Anderson GP, Sundar VC, Bawendi MG (2001). Bioconjugation of Highly Luminescent Colloidal CdSe-ZnS Quantum Dots with an Engineered Two-Domain Recombinant Protein. Phys. Status Solidi B.

[66] Sukhanova A, Venteo L, Devy J, Artemyev M, Oleinikov V, Pluot M, Nabiev I (2002). Highly Stable Fluorescent Nanocrystals as a Novel Class of Labels for Immunohistochemical Analysis of Paraffin-Embedded Tissue Sections. Lab. Invest.

[67] Pathak S, Davidson MC, Silva GA (2007). Characterization of the Functional Binding Properties of Antibody Conjugated Quantum Dots. Nano Lett.

[68] Shepard JRE (2006). Polychromatic Microarrays: Simultaneous Multicolor Array Hybridization of Eight Samples. Anal. Chem.

[69] Rikans LE, Yamano T (2000). Mechanisms of Cadmium-mediated Acute Hepatotoxicity. J. Biochem. Mol. Toxicol.

[70] Alivisatos AP (1996). Perspectives on the Physical Chemistry of Semiconductor Nanocrystals. J. Phys. Chem.

[71] Schroeder JE, Shweky I, Shmeeda H, Banin U, Gabizon A (2007). Folate-mediated Tumor Cell Uptake of Quantum Dots Entrapped in Lipid Nanoparticles. J. Control. Release.

[72] Jayagopal A, Russ PK, Haselton FR (2007). Surface Engineering of Quantum Dots for *In Vivo* Vascular Imaging. Bioconjugate Chem.

[73] Maysinger D, Behrendt M, Lalancette-Hebert M, Kriz J (2007). Real-Time Imaging of Astrocyte Response to Quantum Dots: *In Vivo* Screening Model System for Biocompatibility of Nanoparticles. Nano Lett.

[74] Jiang W, Singhal A, Kim BYS, Zheng J, Rutka JT, Wang C, Chan WCW (2008). Assessing Near-Infrared Quantum Dots for Deep Tissue, Organ, and Animal Imaging Applications. J. Assoc. Lab. Autom.

[75] Zimmer JP, Kim S-W, Ohnishi S, Tanaka E, Frangioni JV, Bawendi MG (2006). Size Series of Small Indium Arsenide-Zinc Selenide Core-Shell Nanocrystals and Their Application to *In Vivo* Imaging. J. Am. Chem. Soc.

[76] Cai W, Shin D-W, Chen K, Gheysens O, Cao Q, Wang SX, Gambhir SS, Chen X (2006). Peptide-Labeled Near-Infrared Quantum Dots for Imaging Tumor Vasculature in Living Subjects. Nano Lett.

[77] Smith JD, Fisher GW, Waggoner AS, Campbell PG (2007). The Use of Quantum Dots for Analysis of Chick CAM Vasculature. Microvasc. Res.

[78] Tada H, Higuchi H, Wanatabe TM, Ohuchi N (2007). *In Vivo* Real-time Tracking of Single Quantum Dots Conjugated with Monoclonal Anti-HER2 Antibody in Tumors of Mice. Cancer Res.

[79] Gopee NV, Roberts DW, Webb P, Cozart CR, Siitonen PH, Warbritton AR, Yu WW, Colvin VL, Walker NJ, Howard PC (2007). Migration of Intradermally Injected Quantum Dots to Sentinel Organs in Mice. Toxicol. Sci.

[80] Ballou B, Ernst LA, Andreko S, Harper T, Fitzpatrick JAJ, Waggoner AS, Bruchez MP (2007). Sentinel Lymph Node Imaging Using Quantum Dots in Mouse Tumor Models. Bioconjugate Chem.

[81] Kobayashi H, Hama Y, Koyama Y, Barrett T, Regino CAS, Urano Y, Choyke PL (2007). Simultaneous Multicolor Imaging of Five Different Lymphatic Basins Using Quantum Dots. Nano Lett.

[82] Bakalova R, Zhelev Z, Aoki I, Masamoto K, Mileva M, Obata T, Higuchi M, Gadjeva V, Kanno I (2008). Multimodal Silica-Shelled Quantum Dots: Direct Intracellular Delivery, Photosensitization, Toxic, and Microcirculation Effects. Bioconjugate Chem.

[83] Chen Z, Chen H, Meng H, Xing G, Gao X, Sun B, Shi X, Yuan H, Zhang C, Liu R (2008). Bio-distribution and Metabolic Paths of Silica Coated CdSeS Quantum Dots. Toxicol. Appl. Pharmacol.

[84] Robe A, Pic E, Lassalle H-P, Bezdetnaya L, Guillemin F, Marchal F (2008). Quantum Dots in Axillary Lymph Node Mapping: Biodistribution Study in Healthy Mice. BMC Cancer.

[85] Liu W, Choi HS, Zimmer JP, Tanaka E, Frangioni JV, Bawendi M (2007). Compact Cysteine-Coated CdSe(ZnCdS) Quantum Dots for *In Vivo* Applications. J. Am. Chem. Soc.

[86] Chang J-C, Su H-L, Hsu S-h (2008). The Use of Peptide-delivery to Protect Human Adipose-derived Adult Stem Cells from Damage Caused by the Internalization of Quantum Dots. Biomaterials.

[87] Liu W, Howarth M, Greytak AB, Zheng Y, Nocera DG, Ting AY, Bawendi MG (2008). Compact Biocompatible Quantum Dots Functionalized for Cellular Imaging. J. Am. Chem. Soc.

[88] Kuo Y-C, Wang Q, Ruengruglikit C, Yu H, Huang Q (2008). Antibody-Conjugated CdTe Quantum Dots for Escherichia coli Detection. J. Phys. Chem. C.

[89] Erogbogbo F, Yong K-T, Roy I, Xu G, Prasad PN, Swihart MT (2008). Biocompatible Luminescent Silicon Quantum Dots for Imaging of Cancer Cells. ACS Nano.

[90] Kairdolf BA, Mancini MC, Smith AM, Nie S (2008). Minimizing Nonspecific Cellular Binding of Quantum Dots with Hydroxyl-Derivatized Surface Coatings. Anal. Chem.

[91] Botsoa J, Lysenko V, Geloen A, Marty O, Bluet JM, Guillot G (2008). Application of 3C-SiC Quantum Dots for Living Cell Imaging. Appl. Phys. Lett.

[92] Yezhelyev MV, Al-Hajj A, Morris C, Marcus AI, Liu T, Lewis M, Cohen C, Zrazhevskiy P, Simons JW, Rogatko A (2007). In Situ Molecular Profiling of Breast Cancer Biomarkers with Multicolor Quantum Dots. Adv. Mater.

[93] Hahn MA, Keng PC, Krauss TD (2008). Flow Cytometric Analysis To Detect Pathogens in Bacterial Cell Mixtures Using Semiconductor Quantum Dots. Anal. Chem.

[94] Uyeda HT, Medintz IL, Jaiswal JK, Simon SM, Mattoussi H (2005). Synthesis of Compact Multidentate Ligands to Prepare Stable Hydrophilic Quantum Dot Fluorophores. J. Am. Chem. Soc.

[95] Mei BC, Susumu K, Medintz IL, Delehanty JB, Mountziaris TJ, Mattoussi H (2008). Modular Poly(ethylene glycol) Ligands for Biocompatible Semiconductor and Gold Nanocrystals with Extended pH and Ionic Stability. J. Mater. Chem.

[96] Susumu K, Uyeda HT, Medintz IL, Pons T, Delehanty JB, Mattoussi H (2007). Enhancing the Stability and Biological Functionalities of Quantum Dots via Compact Multifunctional Ligands. J. Am. Chem. Soc.

[97] Epstein JR, Biran I, Walt DR (2002). Fluorescence-based Nucleic Acid Detection and Microarrays. Anal. Chim. Acta.

[98] Epstein JR, Walt DR (2003). Fluorescence-based Fibre Optic Arrays: A Universal Platform for Sensing. Chem. Soc. Rev.

[99] Sapsford KE, Pons T, Medintz IL, Mattoussi H (2006). Biosensing with Luminescent Semiconductor Quantum Dots. Sensors.

[100] Algar WR, Krull UJ (2008). Quantum Dots as Donors in Fluorescence Resonance Energy Transfer for the Bioanalysis of Nucleic Acids, Proteins, and Other Biological Molecules. Anal. Bioanal. Chem.

[101] Prabhukumar G, Matsumoto M, Mulchandani A, Chen W (2004). Cadmium Removal from Contaminated Soil by Tunable Biopolymers. Environ. Sci. Technol.

[102] Sapsford KE, Pons T, Medintz IL, Higashiya S, Brunel FM, Dawson PE, Mattoussi H (2007). Kinetics of Metal-Affinity Driven Self-Assembly between Proteins or Peptides and CdSe-ZnS Quantum Dots. J. Phys. Chem. C.

[103] Snee PT, Chan Y, Nocera DG, Bawendi MG (2005). Whispering-Gallery-Mode Lasing from a Semiconductor Nanocrystal/Microsphere Resonator Composite. Adv. Mater.

[104] Bentzen EL, Tomlinson ID, Mason J, Gresch P, Warnement MR, Wright D, Sanders-Bush E, Blakely R, Rosenthal SJ (2005). Surface Modification To Reduce Nonspecific Binding of Quantum Dots in Live Cell Assays. Bioconj. Chem.

[105] Pathak S, Choi S-K, Arnheim N, Thompson ME (2001). Hydroxylated Quantum Dots as Luminescent Probes for in Situ Hybridization. J. Am. Chem. Soc.

[106] Wu XL, Fan JY, Qiu T, Yang X, Siu GG, Chu PK (2005). Experimental Evidence for the Quantum Confinement Effect in 3C-SiC Nanocrystallites. Phys. Rev. Lett.

[107] Fan JY, Wu XL, Li HX, Liu HW, Siu GG, Chu PK (2006). Luminescence from Colloidal 3C-SiC Nanocrystals in Different Solvents. Appl. Phys. Lett.

[108] Fan JY, Wu XL, Chu PK (2006). Low-dimensional SiC Nanostructures: Fabrication, Luminescence, and Electrical Properties. Prog. Mater. Sci.

[109] Morris MC, Depollier J, Mery J, Heitz F, Divita G (2001). A Peptide Carrier for the Delivery of Biologically Active Proteins into Mammalian Cells. Nat. Biotechnol.

[110] Fountaine TJ, Wincovitch SM, Geho DH, Garfield SH, Pittaluga S (2006). Multispectral Imaging of Clinically Relevant Cellular Targets in Tonsil and Lymphoid Tissue Using Semiconductor Quantum Dots. Mod. Pathol.

[111] Zhu X-X, Cao Y-C, Jin X, Yang J, Hua X-F, Wang H-Q, Liu B, Wang Z, Wang J-H, Yang L (2008). Optical Encoding of Microbeads Based on Silica Particle Encapsulated Quantum Dots and Its Applications. Nanotechnol.

[112] Zhang Y-h, Zhang H-s, Guo X-f, Wang H (2008). L-Cysteine-coated CdSe/CdS Core-shell Quantum Dots as Selective Fluorescence Probe for Copper(II) Determination. Microchem. J.

[113] Tang B, Cao L, Xu K, Zhuo L, Ge J, Li Q, Lijuan Yu (2008). A New Nanobiosensor for Glucose with High Sensitivity and Selectivity in Serum Based on Fluorescence Resonance Energy Transfer (FRET) between CdTe Quantum Dots and Au Nanoparticles. Chem. Eur. J.

[114] Dickinson ME, Bearman G, Tille S, Lansford R, Fraser SE (2001). Multi-Spectral Imaging and Linear Unmixing Add a Whole New Dimension to Laser Scanning Flouresence Microscopy. BioTechniques.

[115] Wang D, Rogach AL, Caruso F (2002). Semiconductor Quantum Dot-Labeled Microsphere Bioconjugates Prepared by Stepwise Self-Assembly. Nano Lett.

[116] Gao X, Nie S (2003). Doping Mesoporous Materials with Multicolor Quantum Dots. J. Phys. Chem. B.

[117] Cao Y-C, Huang Z-L, Liu T-C, Wang H-Q, Zhu X-X, Wang Z, Zhao Y-D, Liu M-X, Luo Q-M (2006). Preparation of Silica Encapsulated Quantum Dot Encoded Beads for Multiplex Assay and its Properties. Anal. Biochem.

[118] Xie H-Y, Liang J-G, Zhang Z-L, Liu Y, He Z-K, Pang D-W (2004). Luminescent CdSe-ZnS Quantum Dots as Selective Cu2+ Probe. Spectrochim. Acta A.

[119] Fernández-Argüelles MT, Jin WJ, Costa-Fernández JM, Pereiro R, Sanz-Medel A (2005). Surface-modified CdSe Quantum Dots for the Sensitive and Selective Determination of Cu(II) in Aqueous Solutions by Luminescent Measurements. Anal. Chim. Acta.

[120] Jin Y, Lohstreter S, Pierce DT, Parisien J, Wu M, Hall C, Zhao JX (2008). Silica Nanoparticles with Continuously Tunable Sizes: Synthesis and Size Effects on Cellular Contrast Imaging. Chem. Mater.

[121] Medley CD, Smith JE, Tang Z, Wu Y, Bamrungsap S, Tan W (2008). Gold Nanoparticle-Based Colorimetric Assay for the Direct Detection of Cancerous Cells. Anal. Chem.

[122] Park JH, von Maltzahn G, Zhang L, Schwartz MP, Ruoslahti E, Bhatia SN, Sailor MJ (2008). Magnetic Iron Oxide Nanoworms for Tumor Targeting and Imaging. Adv. Mater.

[123] Wang L, Zhang J, Wang X, Huang Q, Pan D, Song S, Fan C (2008). Gold Nanoparticle-based Optical Probes for Target-Responsive DNA Structures. Gold Bull.

[124] Nakamura M, Shono M, Ishimura K (2007). Synthesis, Characterization, and Biological Applications of Multifluorescent Silica Nanoparticles. Anal. Chem.

[125] Gong J-L, Jiang J-H, Yang H-F, Shen G-L, Yu R-Q, Ozaki Y (2006). Novel Dye-embedded Core-shell Nanoparticles as Surface-enhanced Raman Scattering Tags for Immunoassay. Anal. Chim. Acta.

[126] Ow H, Larson DR, Srivastava M, Baird BA, Webb WW, Wiesner U (2005). Bright and Stable Core-Shell Fluorescent Silica Nanoparticles. Nano Lett.

[127] Doering WE, Nie S (2003). Spectroscopic Tags Using Dye-Embedded Nanoparticles and Surface-Enhanced Raman Scattering. Anal. Chem.

[128] Mirkin CA, Letsinger RL, Mucic RC, Storhoff JJ (1996). A DNA-based Method for Rationally Assembling Nanoparticles into Macroscopic Materials. Nature.

[129] Alivisatos AP, Johnsson KP, Peng X, Wilson TE, Loweth CJ, Bruchez MP, Schultz PG (1996). Organization of ‘nanocrystal molecules’ using DNA. Nature.

[130] Lytton-Jean AKR, Han MS, Mirkin CA (2007). Microarray Detection of Duplex and Triplex DNA Binders with DNA-Modified Gold Nanoparticles. Anal. Chem.

[131] Elghanian R, Storhoff JJ, Mucic RC, Letsinger RL, Mirkin CA (1997). Selective Colorimetric Detection of Polynucleotides Based on the Distance-Dependent Optical Properties of Gold Nanoparticles. Science.

[132] Mulvaney SP, Musick MD, Keating CD, Natan MJ (2003). Glass-Coated, Analyte-Tagged Nanoparticles: A New Tagging System Based on Detection with Surface-Enhanced Raman Scattering. Langmuir.

[133] Cao YC, Jin R, Mirkin CA (2002). Nanoparticles with Raman Spectroscopic Fingerprints for DNA and RNA Detection. Science.

[134] Nie S, Emory SR (1997). Probing Single Molecules and Single Nanoparticles by Surface-Enhanced Raman Scattering. Science.

[135] Emory SR, Haskins WE, Nie S (1998). Direct Observation of Size-Dependent Optical Enhancement in Single Metal Nanoparticles. J. Am. Chem. Soc.

[136] Michaels AM, Nirmal M, Brus LE (1999). Surface Enhanced Raman Spectroscopy of Individual Rhodamine 6G Molecules on Large Ag Nanocrystals. J. Am. Chem. Soc.

[137] Moyer PJ, Schmidt J, Eng LM, Meixner AJ, Sandmann GW, Dietz H, Plieth W (2000). Surface-Enhanced Raman Scattering Spectroscopy of Single Carbon Domains on Individual Ag Nanoparticles on a 25 ms Time Scale. J. Am. Chem. Soc.

[138] Moghimi SM, Hunter AC, Murray JC (2001). Long-Circulating and Target-Specific Nanoparticles: Theory to Practice. Pharmacol Rev.

[139] Weissleder R, Bogdanov A, Neuwelt EA, Papisov M (1995). Long-circulating Iron Oxides for MR Imaging. Adv. Drug Delivery Rev.

